# Acute effects of MDMA, MDA, lysine-MDMA, and lysine-MDA in a randomized, double-blind, placebo-controlled, crossover trial in healthy participants

**DOI:** 10.1038/s41386-025-02248-3

**Published:** 2025-09-25

**Authors:** Isabelle Straumann, Patrick Vizeli, Isidora Avedisian, Livio Erne, Diana Noorshams, Ina Vukalovic, Anne Eckert, Dino Luethi, Deborah Rudin, Matthias E. Liechti

**Affiliations:** 1 Clinical Pharmacology and Toxicology, Department of Biomedicine and Department of Clinical Research, University Hospital Basel, Basel, Switzerland; 2 Department of Pharmaceutical Sciences, University of Basel, Basel, Switzerland; 3 Psychiatric University Hospital, University of Basel, Basel, Switzerland; 4 Transfaculty Research Platform Molecular and Cognitive Neuroscience, University of Basel, Basel, Switzerland

**Keywords:** Pharmacology, Translational research

## Abstract

3,4-Methylenedioxymethamphetamine (MDMA) is used recreationally, as a research tool, and in MDMA-assisted therapy in patients with posttraumatic stress disorder. 3,4-Methylenedioxyamphetamine (MDA) is a psychoactive metabolite of MDMA. Acute effects of MDMA and MDA have never been directly compared in humans. Lysine-conjugated amphetamines slowly release active amphetamine once absorbed, suggesting pharmaceutical strategies to enhance tolerability and reduce abuse potential. Therefore, lysine-MDMA (Lys-MDMA) and lysine-MDA (Lys-MDA) were developed as prodrugs of MDMA and MDA, respectively. We used a double-blind, randomized, placebo-controlled, crossover design to compare acute responses to MDMA (100 mg), MDA (92 mg), Lys-MDMA (172 mg), and Lys-MDA (164 mg) at equimolar doses and placebo in 23 healthy participants (12 women, 11 men). Outcome measures included acute subjective, autonomic, and endocrine effects and pharmacokinetics. Compared with placebo, MDMA and MDA produced pronounced subjective and autonomic effects. After Lys-MDMA administration, no MDMA was detected in blood samples, and no corresponding subjective or autonomic effects were observed. MDA produced stronger and longer-lasting subjective “any drug effects” compared with MDMA, with effect durations of (mean ± SEM) 6.1 ± 0.5 vs 4.1 ± 0.4 h, respectively. Additionally, compared with MDMA, MDA induced greater subjective “stimulant effects,” more negative “bad drug effects,” more “fear,” and more “visual alterations.” Lys-MDA, compared with MDA, showed longer times to onset and maximal effect (1.1 ± 0.2 h and 3.0 ± 0.4 h vs. 0.7 ± 0.1 h and 2.0 ± 0.1 h) but otherwise induced similar effects. The plasma elimination half-lives (geometric mean) of MDMA and MDA were 7.3 and 8.4 h, respectively. In summary, MDA produced longer-lasting, stronger, more psychedelic-like perceptual acute effects and more adverse effects compared with MDMA when administered at equimolar doses. Lys-MDA represents a functional slow-release prodrug form of MDA, delaying both the onset and peak of subjective effects. In contrast, Lys-MDMA did not release MDMA, likely because of its tertiary amine structure, and thus does not represent a functional prodrug of MDMA. These results highlight MDA’s less favorable therapeutic profile relative to MDMA and identify lysine conjugation as a potential strategy for modulating, but not necessarily improving, the tolerability of its effects. Trial registration: ClinicalTrials.gov identifier: NCT04847206.

## Introduction

3,4-Methylenedioxymethamphetamine (MDMA) acutely induces feelings of well-being, empathy, trust, closeness, and connectedness [1, 2]. These subjective effects of MDMA are considered helpful to assist psychotherapy for posttraumatic stress disorder [3]. In humans, MDMA is mainly metabolized to the inactive metabolite 4-hydroxy-3-methoxymethamphetamine (HMMA) and the minor but active metabolite 3,4-methylenedioxyamphetamine (MDA) [4]. MDA has previously been investigated for its therapeutic potential in MDA-assisted therapy [5]. Both MDMA and MDA are classified as entactogens or empathogens. Pharmacologically, MDMA and MDA mainly release the monoamines serotonin, norepinephrine, and dopamine [4, 6]. MDA exhibits a greater dopamine-to-serotonin ratio compared with MDMA [6] and thus might have more stimulant-like acute effects than MDMA. MDA is also a serotonin 5-hydroxytryptamine-2A (5-HT_2A_) receptor agonist and exhibits 10-fold higher activation potency at this receptor than MDMA [6]. Because subjective effects of serotonergic psychedelics are mediated by 5-HT_2A_ agonism [7–9], MDA might induce more psychedelic-like effects compared with MDMA. Finally, MDMA is a strong releaser of oxytocin [1, 2, 10, 11], which is thought to contribute to its acute effects on emotion processing [2]. However, it is unknown whether MDA also releases oxytocin. There is limited data on similarities and differences of MDMA’s and MDA’s effects in humans. A small modern study investigated acute effects of MDA (1.4 mg/kg) in 12 healthy human volunteers and compared the results to other studies with MDMA (1.5 mg/kg) [12, 13]. The results indicated comparable acute effects of both substances, with a longer duration of action of MDA (8 h) compared with MDMA (6 h) [12]. Additionally, greater and more psychedelic-like perceptual changes were reported after MDA administration compared with MDMA [12, 13]. However, MDMA’s and MDA’s effects have previously been compared across different studies and participants, but have never been directly and validly compared within the same individuals and study.

Pharmacokinetics of psychoactive substances partly predict their abuse liability and tolerability [14–16]. Specifically, a fast subjective (stimulant or drug-like) effect onset is associated with a higher risk of abuse liability [14–16] but may also be linked with negative acute effects, including anxiety [17]. Therefore, pharmaceutical slow-release formulations or inactive prodrugs with slow liberation of the active substance after absorption have been developed. In the present study, inactive prodrug formulations of MDMA and MDA were developed in the form of lysine-MDMA (Lys-MDMA) and lysine-MDA (Lys-MDA) to potentially slow the onset of acute substance effects, reduce the abuse-related rapid onset of euphoria, reduce possible anxiety at effect onset, and slow the onset of autonomic stimulant effects of the substances. A similar approach has been used with lisdexamfetamine, a comparable marketed inactive lysine-conjugated prodrug formulation of D-amphetamine for the treatment of attention-deficit/hyperactivity disorder [18, 19].

Thus, the present study compared acute responses to equimolar doses of MDMA, MDA, Lys-MDMA, Lys-MDA, and placebo in a double-blind, crossover study in healthy participants. The primary study hypotheses were that (i) MDA would have a longer plasma half-life and produce longer-lasting effects and (ii) induce greater perceptual psychedelic-like effects compared with MDMA. Additionally, we expected that the prodrugs Lys-MDMA and Lys-MDA would show longer times to onset and maximal concentration of the active substance in plasma and associated longer times to effect onset and maximal peak responses compared with MDMA and MDA, respectively. Thus, the prodrug concept was expected to result in a delayed and possibly attenuated acute effect response.

## Methods and materials

### Study design

The study used a double-blind, placebo-controlled, crossover design with five experimental test sessions to investigate responses to (i) placebo, (ii) 100 mg MDMA, (iii) 92 mg MDA, (iv) 172 mg Lys-MDMA, and (v) 164 mg Lys-MDA at equimolar doses. The selected doses correspond to equimolar amounts that are typical doses for MDMA and MDA for clinical or recreational use. Participants were informed that they would receive all treatments. Block randomization was used with counterbalanced treatment order. The washout periods between sessions were at least 14 days. The average time between study days was (mean ± SD) 42 ± 11 days. The study was conducted in accordance with the Declaration of Helsinki and International Conference on Harmonization Guidelines in Good Clinical Practice and approved by the Ethics Committee of Northwest Switzerland and Swiss Federal Office for Public Health. The study was registered at ClinicalTrials.gov (NCT04847206).

### Participants

Twenty-three healthy participants (11 men and 12 women; mean age ± SD: 29 ± 9 years; range: 20–51 years) completed the study and were subsequently analyzed. All subjects provided written informed consent and were paid for their participation. Further details on participants and exclusion criteria are available in the Supplementary Methods.

Prior and current substance use is described in the Supplementary Methods and Supplementary Table S1. Demographics and genetically determined Cytochrome P450 2D6 (CYP2D6) function of the participants are shown in Supplementary Table S2. Genetic determination of CYP2D6 was assessed according to the Clinical Pharmacogenetics Implementation Consortium guidelines [20].

### Study drugs

Racemic MDMA, MDA, Lys-MDMA, and Lys-MDA (ReseaChem, Burgdorf, Switzerland) were administered in gelatin capsules and are described in detail in the Supplementary Methods. Placebo consisted of identical gelatin capsules that were filled with mannitol. The participants received four capsules in each session: (i) four placebo capsules, (ii) four 25 mg MDMA capsules, (iii) four 23 mg MDA capsules, (iv) four 43 mg Lys-MDMA capsules, and (v) four 41 mg Lys-MDA capsules. At the end of each session and at the end of the study, the participants guessed their treatment assignment to evaluate blinding.

### Study procedures

The study included a screening visit, five 13-h test sessions with follow-up measurements 24 h after drug intake, and an end-of-study visit that occurred an average of 28 days after the last test session. The sessions were conducted in a calm hospital room. Only one research participant and one investigator were present in the room during each test session. The test sessions began at 8:00 a.m. A urine pregnancy test was performed in women with childbearing potential. Participants underwent baseline measurements and were questioned to determine exclusion criteria. A standardized breakfast (two croissants) was then served. Substances were administered at 9:00 a.m. The outcome measures were repeatedly assessed for 12 h. Standardized lunches and dinners were served at 1:30 p.m. and 6:00 p.m., respectively. The participants were sent home at 9:15 p.m. and returned the next day for follow-up measurements at 9:00 a.m.

### Subjective drug effects and effect durations

Subjective effects were assessed repeatedly using Visual Analog Scales (VASs) 0.5 h before and 0, 0.25, 0.5, 0.75, 1, 1.5, 2, 2.5, 3, 3.5, 4, 5, 6, 7, 8, 9, 10, 11, 12, and 24 h after drug administration. The VAS item “any drug effect” was used to determine effect onset, offset, time to peak effect, and effect duration.

The Adjective Mood Rating Scale (AMRS) [21] was administered 0.5 h before and 2.5, 5, and 12 h after drug administration. The 5-Dimensions of Altered States of Consciousness (5D-ASC) scale [22] was administered 12 h after drug administration to retrospectively rate peak drug effects. Mystical experiences were assessed 12 h after drug administration using the Psychedelic Experience Scale (PES) [23], a revalidation of the 100-item States of Consciousness Questionnaire [24], which includes the 30-item Mystical Experience Questionnaire [23, 25]. Subjective effect measurements are described in detail in  Supplementary Methods online.

### Autonomic and adverse effects

Blood pressure, heart rate, and tympanic body temperature were measured repeatedly at the same time points as the VAS assessments. Pupil size was assessed at baseline and 1, 2.5, 4, 11, and 24 h after drug administration. Adverse effects were assessed 0.5 h before and 12 and 24 h after drug administration using the List of Complaints [26]. Electrocardiograms were recorded before and 2.5 h after substance administration to determine substance effects on the QTc interval.

### Endocrine effects

Plasma concentrations of oxytocin were measured before and 2, 3, and 6 h after drug administration as previously described [27]. Neurophysin I was measured only for the MDMA, MDA, and placebo conditions in plasma samples using the Oxytocin-neurophysin I prepropeptide in vitro SimpleStep ELISA kit (Abcam, Cambridge, UK) according to the manufacturer’s protocol [28]. Lysine-conjugated substances were expected to exhibit pharmacological profiles for neurophysin I similar to those of their respective parent compounds, as this was controlled for by the oxytocin measurement. Plasma samples were obtained at the same time points as the oxytocin measurements and were collected in lithium heparin tubes.

### Plasma concentrations

Plasma concentrations of MDMA, MDA, and their metabolites were measured repeatedly at the same time points as the VAS assessments (Supplementary Methods).

MDMA, MDA, and their metabolites 4-hydroxy-3-methoxyamphetamine (HMA) and HMMA were analyzed in human plasma using high-performance liquid chromatography tandem mass spectrometry as previously described [29].

### Pharmacokinetic analyses

Pharmacokinetic parameters were estimated using non-compartmental methods as described previously [30] using Phoenix WinNonlin 8.3 (Certara, Princeton, NJ, USA).

### Data analysis

Peak (*E*_max_ and/or *E*_min_) or peak change from baseline (Δ*E*_max_) values were determined from repeated measures between 0 and 12 h post-administration. The study was originally designed as a nested 2 × 2 factorial model, with drug type and lysine-conjugation as factors. However, the factorial design was compromised because Lys-MDMA did not produce detectable MDMA plasma concentrations or effects, rendering it pharmacologically inactive. Consequently, an alternative analytical approach was adopted. Repeated-measures analyses of variance (ANOVAs) were performed to assess the main effect of condition. Upon a significant effect (*p* < 0.05), post hoc analyses with predefined pairwise contrasts were conducted using estimated marginal means, with Holm-adjusted *p* values to account for multiple testing. Five specific comparisons were examined: placebo vs. Lys-MDMA, placebo vs. MDMA, placebo vs. MDA, MDMA vs. MDA, and MDA vs. Lys-MDA. Post-hoc comparisons across all condition levels, when at least one comparison was not predefined, were adjusted using Tukey tests; for example, when comparing subjective drug effects among all pharmacologically active conditions (Table 2) or when comparing genotypes (Table S11) or sex effects (Table S4). Interactions between condition and sex were separately explored by including sex as a between-subjects factor in the model. Effect sizes for within-subject pairwise comparisons were calculated as Cohen’s *d* by dividing the mean of the paired differences by their standard deviation. Effect sizes for the ANOVA were reported as partial eta-squared (*η*_p_^2^). All analyses were conducted using the R language and environment for statistical computing (R 4.4.2, RStudio 2024.12.0). The criterion for significance was *p* < 0.05.

## Results

### Subjective drug effects

**Fig. 1:**
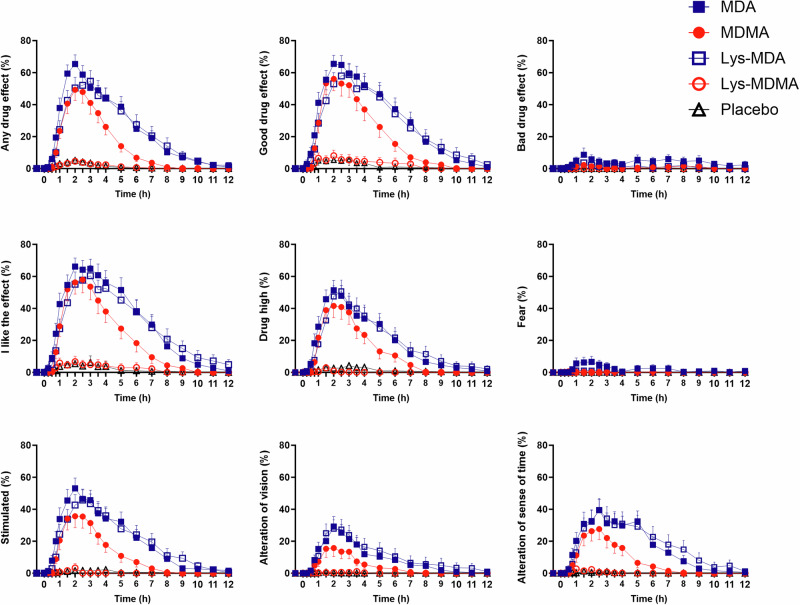
Acute subjective effects of equimolar doses of MDMA, MDA, Lys-MDMA, and Lys-MDA on the Visual Analog Scale (VAS). MDA induced longer lasting and stronger “any drug effects” compared with MDMA. Additionally, MDA induced more stimulation, more negative “bad drug effects,” greater “alteration of vision,” and trendwise greater “alteration of sense of time.” The effect duration of MDA was longer compared with MDMA. Lys-MDA induced effects similar to MDA but showed a slightly later effect onset and a longer time to maximal effect. Lys-MDMA was not psychoactive compared with placebo. The substances were administered at *t* = 0 h. The data are expressed as the mean ± SEM percentage of maximally possible scores in 23 subjects. The corresponding maximal responses and statistics are shown in Table 1.

Subjective effects over time on the VAS are shown in Fig. 1 and Supplementary Fig. S1. Subjective peak responses and statistics are shown in Table 1 and Supplementary Table S3. Characteristics of subjective responses are shown in Table 2. Results of sex × condition interactions are shown in Supplementary Fig. S2 and Supplementary Table S4.

**Table 1:** Mean values and statistics for the acute subjective effects of MDA, MDMA, Lys-MDA, Lys-MDMA, and placebo.

		Placebo	Lys-MDMA	Lys-MDA	MDA	MDMA	*F*_4, 88_	*η*_p_^2^	*p*-value	Pla - Lys-MDMA	Pla - MDA	Pla - MDMA	Lys-MDA -MDA	MDA - MDMA
		mean ± SEM	mean ± SEM	mean ± SEM	mean ± SEM	mean ± SEM								
**Visual Analog Scale (VAS, %max)**														
Unidirectional scales (0–100)														
Any drug effect	*E*_max_	8.6 ± 1.9	5.6 ± 2.1	71 ± 5	73 ± 5	60 ± 6	88.9	0.8	<0.001	0.23	2.54***	1.86***	0.09	0.5*
Good drug effect	*E*_max_	13 ± 5	10 ± 4	73 ± 6	74 ± 5	67 ± 7	60.1	0.73	<0.001	0.1	2.19***	1.62***	0.08	0.26
Bad drug effect	*E*_max_	0 ± 0	0.2 ± 0.2	8.5 ± 2.4	14 ± 4	5.0 ± 2.2	7.2	0.25	<0.001	0.3	0.67***	0.49	0.3	0.51*
I like the effect	*E*_max_	12 ± 5	9.1 ± 4.0	75 ± 6	77 ± 5	68 ± 7	64.4	0.75	<0.001	0.11	2.48***	1.65***	0.08	0.34
Stimulated	*E*_max_	4.6 ± 2.5	5.5 ± 2.9	58 ± 6	62 ± 6	43 ± 7	46.3	0.68	<0.001	0.05	2.15***	1.24***	0.14	0.78**
Drug high	*E*_max_	7.5 ± 3.6	3.9 ± 2.3	60 ± 7	60 ± 6	52 ± 7	43.5	0.66	<0.001	0.17	2.03***	1.45***	0	0.31
Fear	*E*_max_	0.4 ± 0.3	0.1 ± 0.1	3.0 ± 1.3	8.3 ± 3.7	1.6 ± 1.0	3.6	0.14	0.009	0.17	0.43*	0.23	0.36	0.36*
Alteration of vision	*E*_max_	0.9 ± 0.5	2.0 ± 1.1	37 ± 7	38 ± 6	22 ± 5	20.5	0.48	<0.001	0.2	1.3***	0.83**	0.03	0.66*
Alteration of sense of time	*E*_max_	2.7 ± 1.2	3.6 ± 1.9	52 ± 7	53 ± 7	39 ± 7	32.1	0.59	<0.001	0.08	1.59***	1.14***	0.03	0.48
**Autonomic effects**														
Systolic blood pressure (mmHg)	*E*_max_	127 ± 2	128 ± 3	147 ± 3	148 ± 3	146 ± 3	56.6	0.14	<0.001	0.14	2.01***	2.05***	0.08	0.14
Diastolic blood pressure (mmHg)	*E*_max_	77 ± 1	78 ± 1	89 ± 2	89 ± 1	87 ± 2	34.1	0.32	<0.001	0.32	1.63***	1.42***	0.14	0.38
Mean arterial pressure (mmHg)	*E*_max_	93 ± 2	94 ± 2	107 ± 2	108 ± 2	106 ± 2	52.6	0.26	<0.001	0.26	1.9***	1.97***	0.09	0.23
Heart rate (beats/min)	*E*_max_	74 ± 2	77 ± 3	85 ± 3	89 ± 3	92 ± 3	16.3	0.43	<0.001	0.43	1.1***	1.64***	0.25	0.23
Rate pressure product (mmHg x bpm)	*E*_max_	8983 ± 289	9566 ± 408	11966 ± 590	12759 ± 654	13081 ± 578	30.8	0.43	<0.001	0.43	1.42***	1.78***	0.38	0.14
Body temperature (°C)	*E*_max_	37.1 ± 0.05	37.1 ± 0.07	37.6 ± 0.09	37.4 ± 0.04	37.3 ± 0.07	12.6	0.03	<0.001	0.03	1.06***	0.67*	0.32	0.32
Pupil size (mm)	E_max_	6.1 ± 0.2	6.1 ± 0.2	7.1 ± 0.2	7.2 ± 0.2	7.0 ± 0.2	63.2	0.78	<0.001	0.12	2.16***	2.48***	0.28	0.5
Pupil size after light (mm)	E_max_	4.4 ± 0.2	4.3 ± 0.2	6.0 ± 0.2	6.2 ± 0.2	5.9 ± 0.2	153.6	0.88	<0.001	0.18	3.32***	2.82***	0.54	0.68*
Pupil contraction (mm)	E_min_	1.5 ± 0.1	1.5 ± 0.1	1.0 ± 0.1	0.9 ± 0.1	1.0 ± 0.1	92.7	0.81	<0.001	0.25	2.4***	1.88***	0.47	0.31
**List of Complaints (ΔLC Score)**														
Acute adverse effects	0–12 h	1.1 ± 0.5	0.6 ± 0.8	11 ± 2	12 ± 2	5.8 ± 1.0	31.6	0.59	<0.001	0.17	1.35***	1.09**	0.42	0.98***
Subacute adverse effects	12–24 h	−0.7 ± 0.4	−0.5 ± 0.7	6.0 ± 1.4	5.0 ± 1.1	0.6 ± 0.6	15.7	0.42	<0.001	0.08	1.01***	0.44	0.18	0.83**
**Hormones and Markers**														
Oxytocin (pg/mL)	Δ2 h	−38 ± 16	1.6 ± 8.6	195 ± 39	346 ± 58	239 ± 51	23.5*α*	0.53	<0.001	0.5	1.62***	1.11***	0.76**	0.35
	Δ3 h	−43 ± 16	4.2 ± 12	330 ± 53	312 ± 50	240 ± 48	25.1*α*	0.54	<0.001	0.45	1.67***	1.41***	0.1	0.32
	Δ4 h	−34 ± 16	−0.8 ± 9	360 ± 52	287 ± 53	206 ± 44	23.3*β*	0.54	<0.001	0.4	1.22***	1.22***	0.24	0.47
	Δ6 h	−38 ± 15	−12 ± 11	204 ± 35	156 ± 36	100 ± 43	16.7*γ*	0.29	<0.001	0.42	1.01***	0.67**	0.37	0.37
	Δ*C*_max_	−14 ± 14	27 ± 10	452 ± 46	458 ± 53	341 ± 47	50.9*γ*	0.65	<0.001	0.44	1.82***	1.46***	0.06	0.37*
Neurophysin I (pg/mL)	Δ2 h	−23 ± 18			12,037 ± 1971	10,155 ± 1819	32.1δ	0.59	<0.001		1.28***	1.17***		0.46
	Δ3 h	−58 ± 37			11,807 ± 1409	11,021 ± 1801	40.5δ	0.65	<0.001		1.77***	1.29***		0.14
	Δ4 h	−33 ± 30			10,441 ± 1158	8561 ± 1435	40.7δ	0.65	<0.001		1.89***	1.25***		0.36
	Δ6 h	0.9 ± 32			6099 ± 766	4159 ± 863	35.2δ	0.62	<0.001		1.65***	1***		0.73*
	Δ*C*_max_	59 ± 29			14,532 ± 1759	12,376 ± 1827	50.5*δ*	0.7	<0.001		1.72***	1.41***		0.48

*η*_p_^2^, parial eta-squared; post-hoc comparisons display Cohen’s *d* and *p-*value*:* *p < 0.05, **p < 0.01, ***p < 0.001; *Δ* effect difference from baseline; *α*, *F*(4,64); *β*, *F*(4,72); *γ*, *F*(4,76); *δ*, *F*(2,44); *N* = 23.

**Table 2:** Parameters characterizing the subjective drug effect-time curves of MDMA, MDA, and Lys-MDA.

	MDMA 100 mg	MDA 92 mg	Lys-MDA 164 mg	*F*_2,40_	*η*_p_^2^	*p*-value	Lys-MDA - MDA	Lys-MDA - MDMA	MDA - MDMA
Time to onset (h)	0.9 ± 0.1 (0.4–1.6)^α^	0.7 ± 0.1 (0.3–1.1)	1.1 ± 0.2 (0.2–3.3)^β^	3.4	0.14	0.045	0.54*	0.14	0.51
Time to offset (h)	4.6 ± 0.4 (1.4–7.4)^α^	6.8 ± 0.5 (1.7–11)	7.0 ± 0.6 (2.3–12)^β^	12.7	0.39	<0.001	0.06	1.07***	0.92***
Time to maximal effect (h)	2.0 ± 0.2 (0.8–3.5)	2.0 ± 0.1 (1.0–3.5)	3.0 ± 0.4 (1.0–8.0)	6.2^γ^	0.22	0.004	0.51*	0.59**	0.03
Effect duration (h)	4.1 ± 0.4 (1.8–6.7)^α^	6.1 ± 0.5 (0.9–10)	6.0 ± 0.5 (1.2–11)^β^	13.0	0.39	<0.001	0.07	1.2***	1.09***
Maximal effect (%)	60 ± 6 (6–100)	73 ± 5 (19–100)	71 ± 5 (9–100)	4.8^γ^	0.18	0.013	0.09	0.62*	0.5*
AUEC (h*%)	163 ± 24 (8–455)	316 ± 38 (42–671)	288 ± 39 (12–702)	14.1^γ^	0.39	<0.001	0.17	0.98***	1.05***

Parameters are for “any drug effects”. The threshold to determine times to onset and offset was set at 10%. Values are mean ± SEM (range). Posthoc comparisons display Cohen’s *d* and *p*-value: *p < 0.05, ***p < 0.001; *N* = 23, ^α^*N* = 20, ^β^*N* = 22, ^γ^*F*_2,44._

MDA produced longer-lasting and greater “any drug effects” compared with MDMA. Additionally, MDA induced more “bad drug effects” and greater subjective stimulation, fear, and visual changes compared with MDMA on the VAS. On the PES48, MDA produced significantly higher ratings of “distressing experience” and nominally higher “visual experience” ratings compared with MDMA (Supplementary Fig. S3, Supplementary Table S5). All other subjective effects were comparable. On the 5D-ASC, MDA induced nominally more visual changes on the “complex imagery” and “elementary imagery” subscales, but no significant differences were found (Supplementary Fig. S4, Supplementary Table S6). On the ARMS, MDA induced more emotional excitation compared with MDMA (Supplementary Fig. S5, Supplementary Table S7).

Both MDA and MDMA reached *E*_max_ after 2 h, but the effect duration of MDA was 2 h longer (6.1 ± 0.5 h; mean ± SEM) compared with MDMA (4.1 ± 0.4 h; Table 2).

Lys-MDA did produce a significantly delayed onset of effect (1.1 ± 0.2 vs. 0.7 ± 0.1 h) and a significantly longer time to peak effect (3.0 ± 0.4 vs. 2.0 ± 0.1 h; Table 2).

### Autonomic and adverse effects

**Fig. 2:**
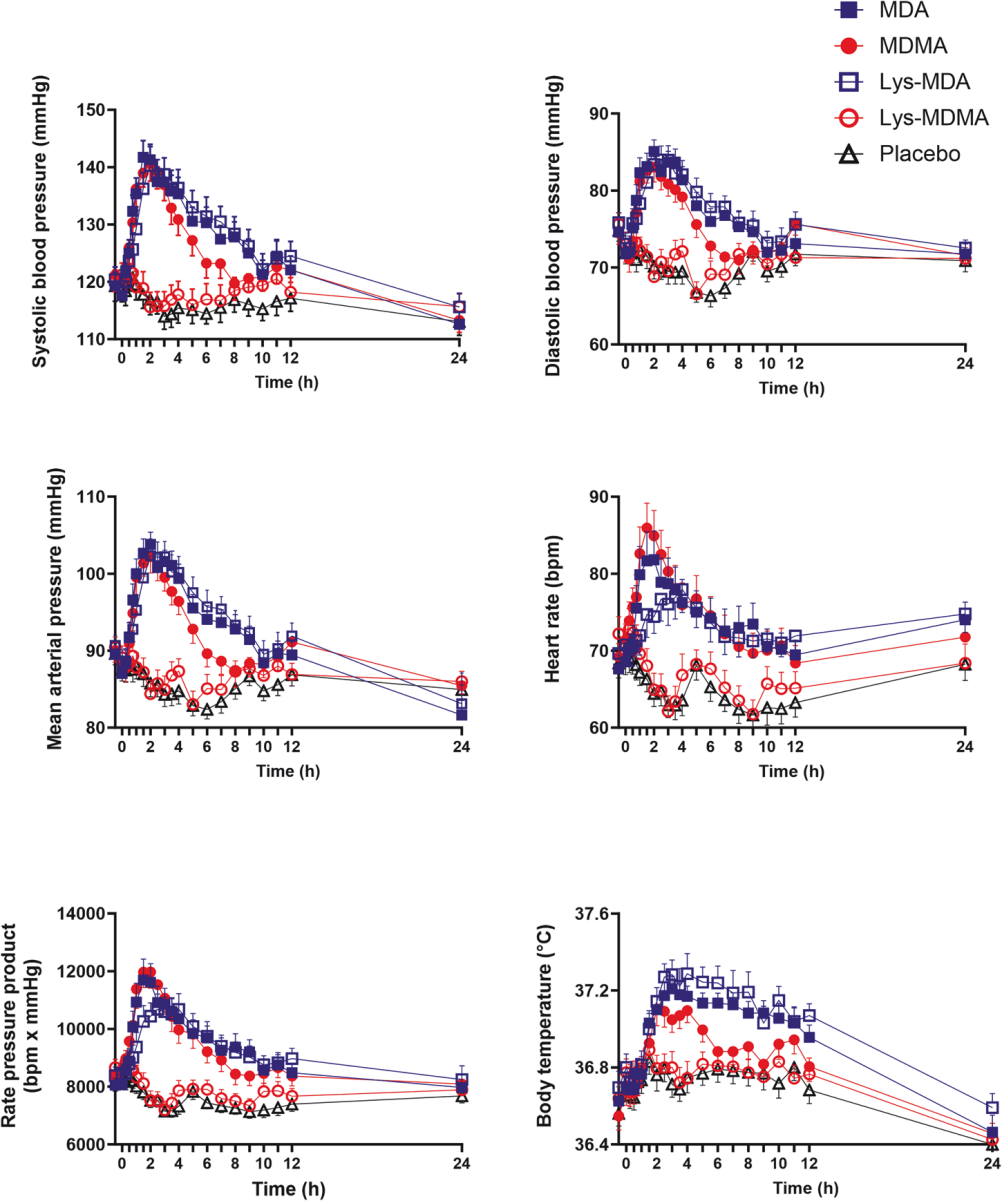
Acute autonomic effects of equimolar doses of MDMA, MDA, Lys-MDMA, and Lys-MDA. MDMA, MDA, and Lys-MDA induced similar increases in blood pressure, heart rate, and body temperature compared with placebo. Lys-MDMA was inactive. The substances were administered at *t *= 0 h. The data are expressed the mean ± SEM in 23 subjects. The corresponding maximal responses and statistics are shown in Table 1.

Autonomic effects over time and related peak responses are shown in Fig. 2 and Table 1, respectively.

MDMA, MDA, and Lys-MDA produced comparable increases in blood pressure, heart rate, body temperature, and pupil diameter. However, effects of MDA and Lys-MDA were longer-lasting compared with MDMA. MDA produced marginally greater pupil dilation following light exposure than MDMA (Supplementary Fig. S6). Lys-MDMA did not produce any changes in blood pressure, heart rate, body temperature, or pupil size.

MDA and Lys-MDA produced significantly more acute and subacute adverse effects on the List of Complaints compared with MDMA (Table 1). More subacute adverse effects after MDA and Lys-MDA were also reported by female participants compared to male participants and by participants who were genetically determined poor metabolizers (PMs) of CYP2D6 compared to intermediate (IMs) and extensive metabolizers (EMs). Frequently reported adverse effects are listed in Supplementary Table S8. No substance changed the QTc interval (Supplementary Table S9).

### Effects on circulating oxytocin and neurophysin I

MDMA, MDA, and Lys-MDA significantly increased plasma oxytocin concentrations compared with placebo. Lys-MDA increased oxytocin levels later than MDA (Table 1 and Supplementary Fig. S7). MDA and MDMA markedly increased plasma levels of neurophysin I compared with placebo. MDA increased plasma levels of neurophysin I slightly more than MDMA. Oxytocin and neurophysin I concentrations were strongly intercorrelated, with every 1000 pg/ml increase in neurophysin I corresponding to an increase in oxytocin of 28 pg/ml (Pearson correlation, *r* = 0.82, *p* < 0.001; Supplementary Fig. S8).

### Plasma drug concentrations

**Fig. 3:**
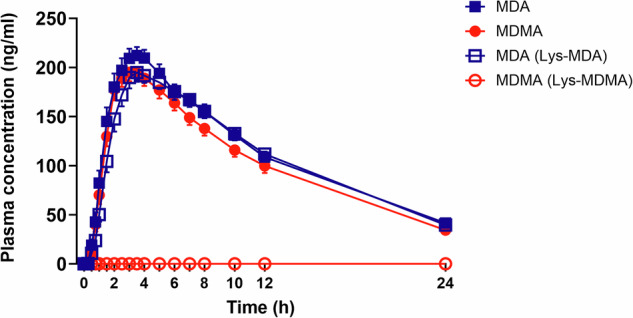
Plasma concentration of MDMA and MDA after the administration of equimolar doses of MDMA, MDA, Lys-MDMA, and Lys-MDA. The maximal concentration (*C*_max_) of MDMA was 208 ng/ml after the administration of MDMA. *C*_max_ values of MDA were 231 and 207 ng/ml after MDA and Lys-MDA administration, respectively. The time to maximal plasma concentration (*t*_max_) of MDMA was 3.0 h. *t*_max_ values of plasma MDA were 3.4 and 3.8 h after MDA and Lys-MDA administration, respectively. Plasma elimination half-lives were 7.3, 8.4, and 7.9 h after MDMA, MDA, and Lys-MDA administration, respectively. No MDMA could be measured after the administration of Lys-MDMA. The substances were administered at *t* = 0 h. The data are expressed as the geometric mean ± SEM. Statistics are shown in Supplementary Table S10.

Drug concentration–time curves are shown in Fig. 3 and Supplementary Figs. S9 and S10. Pharmacokinetic parameters are shown in Supplementary Table S10. Average elimination half-lives (*t*_1/2_) for MDMA, MDA, and MDA after Lys-MDA administration were 7.3, 8.4, and 7.9 h, respectively. Times to maximal concentration (*t*_max_) values were 3.0, 3.4, and 3.8 h after MDMA, MDA, and Lys-MDA administration, respectively. The maximal MDA concentration was slightly higher after MDA administration (231 ng/ml) compared with Lys-MDA (207 ng/ml; Supplementary Table S10). After Lys-MDMA administration, no MDMA was measured in plasma. Area under the curve (AUC) was increased and *t*_1/2_ longer in PMs of CYP2D6 compared to IMs and EMs after the administration of MDMA. When MDA and Lys-MDA were administered, *t*_1/2_ of MDA was longer in PMs compared to IMs and EMs. Additionally, *C*_max_ and AUC of the main metabolites HMMA and HMA were increased in PMs compared to IMs and EMs, and in IMs compared to EMs (Supplementary Table S11). Plasma concentrations of MDMA and MDA over time in different CYP2D6 metabolizers are shown in Supplementary Fig. S11. Area ratios of Lys-MDA and Lys-MDMA are shown in Supplementary Fig. S12.

### Blinding

The participants could not distinguish effects of MDMA, MDA, and Lys-MDA (Supplementary Table S12) after the treatment sessions or at the end-of-study visit. Placebo was correctly identified by 74% of participants after the study session. Lys-MDMA was misclassified as placebo by 65% of participants after the study session.

### Placebo effects

When comparing the placebo and Lys-MDMA sessions (with Lys-MDMA serving as a second unexpected placebo session), the participants reported greater subjective effects during the second, later-occurring session regardless of the actual drug condition (Supplementary Table S13).

## Discussion

The present study was the first to directly compare the acute effects of MDMA and MDA in the same healthy participants. MDA produced longer-lasting acute effects than MDMA. MDA also induced more perceptual hallucinogen-like changes than MDMA, including significantly higher VAS ratings of “alteration of vision” and nominally greater increases in “complex imagery” and “elementary imagery” on the 5D-ASC. Furthermore, MDA induced greater subjective stimulation, more negative “bad drug effects,” and more acute adverse effects compared with MDMA. Subacute adverse effects, including headache, lack of energy, lack of appetite, increased need to sleep, and feeling exhausted, were also more frequently reported after MDA compared with MDMA.

The present finding of longer acute effects of MDA compared with MDMA is consistent with a previous study of MDA’s effects, including a comparison with MDMA data from another study [12]. The longer effect duration of MDA compared with MDMA is likely attributable to the longer plasma elimination half-life of MDA compared with MDMA.

The greater stimulant-type effects of MDA compared with MDMA are consistent with the more stimulant-like in vitro pharmacological profile of MDA compared with MDMA [6]. Specifically, the dopamine-to-serotonin transporter inhibition potency ratio values are 0.24 and 0.14 for MDA and MDMA, respectively, consistent with a slightly more dopaminergic vs. serotonergic activation profile for MDA vs. MDMA [6]. Similarly, the greater perceptual psychedelic-like properties of MDA can be explained by its 10-fold higher potency as a 5-HT_2A_ receptor agonist [6], which mediates subjective effects of classic serotonergic psychedelics [7–9]. However, these more pronounced psychedelic-like effects were not significantly reflected in the psychedelics-focused 5D-ASC questionnaire, suggesting that MDA remains more closely aligned with MDMA than with classic psychedelics such as LSD or psilocybin.

The present study was the first to assess plasma oxytocin levels after MDA administration. MDMA has repeatedly been shown to strongly increase oxytocin levels, and oxytocin is also thought to contribute to the acute subjective effects of MDMA, including feeling more open, close to others, and trusting [2]. Interestingly, in the present study, MDA increased circulating levels of oxytocin even more and slightly longer than MDMA. Both MDA and MDMA also strongly increased plasma levels of neurophysin I, which is a carrier protein of oxytocin and marker for oxytocin activation. Oxytocin levels correlated well with neurophysin I levels, supporting neurophysin I as an accurate biomarker that might be more stable and easier to measure than oxytocin [28]. The release of oxytocin following MDMA administration has been discussed as a key mechanism that contributes to its therapeutic effects in PTSD patients, including fear extinction learning [30–33]. In the present study, MDA increased oxytocin levels at least as much as MDMA and produced comparable effects on the VAS ratings of “trust,” “feeling close to others,” “open,” and “talkative.” These findings indicate that MDA may share certain prosocial effects with MDMA that could be of potential interest for substance-assisted therapy. However, the present results also show that MDA had a less favorable tolerability profile and was associated with a slightly more negative overall experience, including higher ratings of “bad drug effects” and “fear” on the VAS, higher scores on the PES48 “distressing experience” subscale, and more reports of acute and subacute adverse effects on the List of Complaints, compared with MDMA. These factors may limit its suitability for use in substance-assisted therapy. However, despite the small differences in pharmacokinetics and pharmacodynamics between MDA and MDMA, their autonomic effects did not differ significantly, and neither substance altered the ECG QTc interval, which is consistent with previous reports for MDMA [3].

The present study allowed testing sex differences in the response to MDMA, which have previously been reported and typically include greater acute effects in women than in men at similar doses [34–37]. In the present study, we found largely similar acute subjective effects among female and male participants. However, females had higher ratings of “alteration of vision” and “alteration of sense of time” on the VASs. Female participants also reported more acute and subacute adverse drug effects after MDA and Lys-MDA administration than male participants. The cause of these sex differences remains unclear. When body weight was included as a covariate, the observed interactions between sex and condition persisted, indicating that sex effects were not driven by weight differences between sexes, in contrast to previous studies [37].

MDMA is metabolized primarily by CYP2D6, and differences in genetic polymorphisms on pharmacokinetics have been shown before [38]. In the present study, *t*_1/2_ was longer and plasma AUC greater in PMs compared to IMs and EMs after MDMA administration. PMs also showed an increased *t*_1/2_ of MDA when MDA or Lys-MDA was administered. *C*_max_ and AUC of HMMA or HMA followed a genotype-dependent pattern, with CYP2D6 genotype associated with higher enzymatic activity corresponding to higher HMMA or HMA levels. CYP2D6 genotypes had no effect on subjective effects, but PMs reported significantly more subacute adverse effects compared to IMs and EMs.

Lys-MDA and Lys-MDMA were developed and tested in the present study as prodrug slow-release formulations of MDA and MDMA, respectively. The use of prodrugs in the present study sought to reduce abuse-related rapid-onset euphoria, sharp increases in blood pressure, and anxiety at effect onset. In particular, the rapid delivery of amphetamines is considered an important predictor of abuse liability [39, 40], and a slow rise of the blood concentration, which is usually observed with extended-release formulations, is associated with delayed and lower subjective effects, potentially resulting in lower abuse potential [39]. The lysine-amphetamines are inactive and expected to be gradually converted into their active forms in the circulation. The same prodrug approach has been implemented with the marketed inactive prodrug lisdexamfetamine, which consists of lysine- and D-amphetamine and releases active D-amphetamine in the circulation [14, 19]. Consistent with our hypothesis, the effect onset of Lys-MDA was slightly later, and the time to maximal effect was delayed by 1 h after Lys-MDA administration compared with MDA. MDA and Lys-MDA produced similar maximal increases in blood pressure, heart rate, body temperature, and pupil size, but autonomic stimulation was delayed after Lys-MDA administration compared with MDA. Lys-MDA and MDA induced overall comparable subjective effects on the VAS, AMRS, 5D-ASC, and PES, except for a higher rating of “distressing experience” on the PES48 after MDA administration compared with Lys-MDA.

Lys-MDA also increased oxytocin levels, similarly to MDA, but also with a delay compared with MDA. Altogether, the effect onset of Lys-MDA was slightly attenuated compared with MDA, and Lys-MDA may have some benefits over MDA. In contrast, Lys-MDMA did not induce any subjective or autonomic effects, and no MDMA was detected in the blood plasma samples. Lys-MDMA has a tertiary amine structure in contrast to MDA, which is a secondary amine. The tertiary amine structure likely prevents metabolism by plasma peptidases, and thus, the active compound was not released. Consequently, Lys-MDMA does not function as an effective prodrug of MDMA, whereas Lys-MDA and lisdexamfetamine function as effective prodrugs of MDA and amphetamine, respectively.

We did not expect Lys-MDMA to have no subjective effects. Therefore, Lys-MDMA served as a second unexpected placebo session and provided an opportunity to investigate the placebo effect in a clinical study with psychoactive substances. Specifically, the participants and investigators knew beforehand that the study included only one placebo session. However, the second session without any drug effects was not anticipated. Interestingly, participants’ ratings of drug intensity were significantly higher in these second “unexpected placebo” sessions compared with the first “expected placebo” sessions. Peak ratings of “any drug effects,” “good drug effects,” “I like the effect,” and “stimulated” increased with a trend toward higher ratings of “drug high” during the second compared with the first placebo session. These effects were more pronounced at the beginning of the session when participants anticipated substance effects. This finding is consistent with an expectancy effect and highlights the importance of accounting for expectancy effects in clinical trials that involve psychoactive substances, particularly when using within-subjects designs. The finding also underscores that the strong subjective effects that are commonly observed in psychedelic research may limit the interpretive value of an a priori-known placebo control session.

The present study is the first to comprehensively describe the pharmacokinetics of MDA and include a direct comparison with MDMA. We used a fixed dose of 92 mg MDA hydrochloride, which is mole-equivalent to 100 mg MDMA hydrochloride. The maximal plasma concentration of MDA in the present study was 231 ng/ml (geometric mean) and similar to the maximal concentration of 229 ng/ml that was reported in a previous study that used body weight-adjusted doses of 1.4 mg/kg [12]. The elimination half-life of 8.4 h for MDA was shorter in the present study than the previously reported value of 10.9 h [12]. These differences could be attributable to differences in study methods and participants.

The present study has several strengths. This study is, to our knowledge, the first to investigate an MDMA-like substance and directly compare it with MDMA in humans. While other analogous and prodrugs have been proposed [41, 42], none have been tested clinically. The study employed a relatively large sample size (*n* = 23), and statistically powerful within-subjects comparisons were used in a randomized, double-blind design. A wide range of internationally established psychometric outcome measures was used. Plasma concentrations were determined at close intervals in all participants and analyzed with validated methods. Additionally, an unexpected placebo condition allowed comparisons of order-related placebo and expectancy effects in a clinical study with substances that are anticipated to have psychoactive effects.

Notwithstanding these strengths, the present study also has several limitations. Due to the unexpected lack of pharmacological activity exhibited by Lys-MDMA, the initial statistical analysis plan could not be implemented. Additionally, we administered only a single dose level of the substances, using the racemic mixtures, which is the form used in both clinical and recreational use. Enantiomers of MDMA may produce slightly distinct effects [37]. Furthermore, although the present study was conducted in a highly controlled hospital setting with healthy volunteers, involved only a limited number of doses, and included adequate washout periods, repeated MDMA or MDA administration, whether in recreational or clinical contexts, may alter pharmacokinetics, increase conversion to MDA, and carry potential risks of serotonergic neurotoxicity [43] and dependence [44]. These factors should be carefully considered in therapeutic applications. Moreover, individuals in different environments or patients with psychiatric disorders may respond differently to these substances. Finally, the outcome measures might not have been sufficiently sensitive to capture all aspects of the substance experience and very subtle differences between acute effects of MDMA and MDA.

## Conclusion

In the present study, MDA produced longer-lasting and stronger subjective drug effects, greater subjective stimulation, and more bad drug effects and fear. Moreover, MDA elicited more pronounced psychedelic-like perceptual effects and was associated with more acute and subacute adverse effects compared with MDMA. Lys-MDA functioned as a slow-release prodrug of MDA, delaying both the onset and peak effects. Overall, the results suggest a narrower therapeutic margin for MDA compared with MDMA and indicate that lysine conjugation of MDA may be a viable strategy to modify the time course of effects, though not necessarily to enhance overall tolerability.

## Supplementary information

Supplement

## Data Availability

The datasets presented in this article are not readily available because the data associated with this work are owned by the University Hospital Basel and were licensed by Mind Medicine, Inc. Requests to access the datasets should be directed to MEL, matthias.liechti@usb.ch.
