# Supplementary material for: Acute effects of MDMA, MDA, lysine-MDMA, and lysine-MDA in a randomized, double-blind, placebo-controlled, crossover trial in healthy participants
Source: Neuropsychopharmacology. 2025 Sep 25;51(2):476–85. doi: 10.1038/s41386-025-02248-3 (PMC12708835; doi:10.1038/s41386-025-02248-3)
Supplement: Supplementary file 1 — Supplement [file 41386_2025_2248_MOESM1_ESM.docx]

**Supplement**

**Methods**

*Screening Procedure*

Prior to screening, subjects receive written information about the study’s procedures and risks, and informed consent is obtained by the investigator or coordinator on the screening day, with a study physician available for medical questions. The participant information covers the study’s purpose, procedures, duration, risks, benefits, and participants’ rights, including voluntary participation and the option to withdraw at any time without affecting future care. Subjects are given at least 24 hours to decide and must sign the approved consent form before any study procedure starts.

At screening, physical health is assessed by a study physician through medical history, physical exam, ECG, body weight, and blood and hematology analyses. Mental health screening uses a semi-structured DSM-IV interview [1] and the PQ-16 [2]. Participants with psychiatric findings receive guidance on professional help.

*Participants and exclusion criteria*

Thirty-nine individuals were recruited by word of mouth or from a pool of volunteers who had contacted our research group because they were interested in participating in a clinical trial on psychedelics or entactogens. Twenty-six participants met the criteria for study inclusion and were randomized. There were three drop-outs after the first study session. Exclusion criteria were < 18 years or > 65 years of age, pregnancy (urine pregnancy test at screening and before each test session), personal or family (first-degree relative) history of major psychiatric disorders (assessed by the Semi-structured Clinical Interview for *Diagnostic and Statistical Manual of Mental Disorders*, 5^th^ edition, Axis I disorders), the use of medications (e.g., antidepressants, antipsychotics, sedatives, or other compounds) that may interfere with the study medications, chronic or acute physical illness (e.g., abnormal physical exam, electrocardiogram (ECG), or hematological and chemical blood analyses), tobacco smoking (> 10 cigarettes/day), lifetime prevalence of MDMA use > 20 times, illicit drug use within the last 2 months (except for Δ^9^-tetrahydrocannabinol [THC]) and during the study period (including THC). For each subject, urine drug screens were conducted at the screening and randomly at least once before a study day, excluding those positive for stimulants, opioids, or tranquilizers. The participants were asked to consume no more than 20 standard alcoholic drinks/week and have no more than one drink on the day before the test sessions. Participants were required to abstain from all medications, over-the-counter substances, and plant products that could affect study outcomes, as confirmed by self-report prior to each session.

*Prior and current substance use*

We aimed at including persons with no or limited previous drug experience as similar substance use experiences are mostly observed in patients treated with MDMA. Thus, persons with no prior experience were included as well as persons with a few prior experiences. However, we excluded more experienced persons with > 20 prior illicit substance uses. There was no restriction on prior use of Δ^9^-tetrahydrocannabinol (THC) as THC-use is prevalent. However, persons with any substance use disorder including THC were excluded.

Seven participants had previously used MDMA (1–15 times), 10 participants had used a psychedelic (1–12 times), and eight participants had used a stimulant, including cocaine (three participants, 1–20 times), amphetamine (three participants, 1–4 times), and methylphenidate (five participants, 1–10 times). Three participants had used nitrous oxide (5–100 times), three participants had used ketamine (3–7 times), and two participants had used a benzodiazepine (1–7 times). Eight participants had never used any illicit drugs, with the exception of THC. One participant smoked two tobacco cigarettes daily, two participants smoked one tobacco cigarette daily and one participant smoked occasionally. Twenty participants drank alcohol. Mean ± SD consumption of alcohol was 2.7 ± 2.6 standard drinks per week (range: 1-11). Twenty participants had used THC (Table S1).

*Study drugs*

MDMA (ReseaChem, Burgdorf, Switzerland) was administered in gelatin capsules that contained 25 mg MDMA hydrochloride (HCl) with an exact analytically confirmed actual MDMA HCl content of 24.27 ± 0.57 mg (*n* = 10 samples). MDA (ReseaChem, Burgdorf, Switzerland) was administered in gelatin capsules that contained 23 mg MDA HCl and an exact analytically confirmed actual MDA HCl content of 23.75 ± 1.26 mg (*n* = 10 samples).

Lys-MDMA (ReseaChem, Burgdorf, Switzerland) was administered in gelatin capsules that contained 43 mg Lys-MDMA dihydrochloride. Lys-MDA (ReseaChem, Burgdorf, Switzerland) was administered in gelatin capsules that contained 41 mg Lys-MDA dihydrochloride. Placebo consisted of identical gelatin capsules that were filled with mannitol. All capsules were produced according to Good Manufacturing Practice guidelines (Dr. Hysek AG, Biel, Switzerland).

*Subjective drug effects measurements*

*Visual Analog Scales (VASs)*

Subjective effects were assessed repeatedly using visual analog scales (VASs) [3,4] 0.5 h before and 0, 0.25, 0.5, 0.75, 1, 1.5, 2, 2.5, 3, 3.5, 4, 5, 6, 7, 8, 9, 11, 12, and 24 h after drug administration. The VASs included “any drug effect”, “good drug effect”, “bad drug effect”, “I like the effect”, “stimulated”, “drug high”, “fear”, “alteration of vision”, and “alteration of sense of time” that were presented as 100-mm horizontal lines (0-100%), marked from “not at all” on the left to “extremely” on the right [3,5]. Further VASs included “emotional”, “happy”, “talkative”, “open”, “trust”, “I feel close to others” “I want to be alone”, and “I want to be with others”. These VASs were bidirectional and marked with “normal” in the middle at 0 mm and “not at all” (-50 mm) on the left and “extremely” (50 mm) on the right. The primary VAS outcome measures were t_onset_, t_max_, E_max_, and AUEC of the “any drug effect”. The VASs included in the present study have been repeatedly used and shown to be sensitive to the acute effects of MDMA [3,5-7]. Additionally, the VAS “alteration of vision” and “alteration of sense of time” were included because ratings on them were shown to be increased after the administration of different psychedelics [7-9] and to better capture potentially psychedelic-like effects of MDMA and MDA in the present study. The VASs can be completed relatively rapidly and easily by the participant even during the MDMA experience and allow for a valid real-time evaluation of the drug effects over time. The VASs are sensitive and relatively simple measures. More complex assessments of the state of MDMA have to be performed primarily at the end of the session and include entire multi-item questionnaires as described below. The VAS “any drug effect” is an overall effect measure to characterize the overall substance effect intensity and time course. The VAS “good drug effect” is an overall measure of effects subjectively considered positive and interrelated with other measures such as “drug liking”. The VAS “bad drug effect” is an overall measure of any negative effects and is related to “fear”.

Maximal effect (E_max_), time to effect onset (t_onset_), time to maximal effect, effect duration, and area under the effect curve (AUEC) were assessed using Phoenix WinNonlin 8.3 (Certara, Princeton, NJ, USA) and “any drug effect” VAS effect-time plots and an onset/offset threshold of 10% of the maximum possible response. Participants with responses < 15% were not used to determine the time to onset, time to offset, or effect duration.

*Adjective Mood Rating Scale (AMRS)*

The Adjective Mood Rating Scale (AMRS) [10] was used 0.5 h before and 2.5, 5, and 12 h after drug administration. The AMRS is a validated 60-item Likert mood rating scale mainly used in Europe and consists of subscales including ratings on “well-being”, “anxiety”, “inactivity”, “extraversion”, “introversion”, and “emotional excitation”. It is suitable for repeated measurements of mood states. The short German EWL60S version was used [10]. The completion of the ratings under the effects of psychedelic substances is possible but difficult because it lasts several minutes. The scale was used in paper and pencil version, but it may be more suitable to use this measure verbally during states of markedly impaired concentration. The AMRS was included as a secondary measure because it could be considered a better validated measure of mood states and producing more defined ratings than the VAS and to support findings on the VAS (AMRS well-being considered similar to VAS good drug effects; AMRS anxiety considered similar to VAS fear).

*5 Dimension of Altered States of Consciousness (5D-ASC) scale*

The 5 Dimensions of Altered States of Consciousness (5D-ASC) scale [11,12] was administered 12 h after drug administration to retrospectively rate peak drug effects. The 5D-ASC scale measures altered states of consciousness and contains 94 items (visual analog scales). The instrument consists of five subscales/dimensions [11] and 11 lower-order scales [12]. The 5D-ASC dimension “Oceanic Boundlessness” (27 items) measures derealization and depersonalization associated with positive emotional states, ranging from heightened mood to euphoric exaltation. The corresponding lower-order scales include “experience of unity,” “spiritual experience,” “blissful state,” “insightfulness,” and “disembodiment.” The dimension “Anxious Ego Dissolution” (21 items) summarizes ego-disintegration and loss of self-control phenomena associated with anxiety. The corresponding lower-order scales include “impaired control of cognition” and “anxiety.” The dimension “Visionary Restructuralization” (18 items) consists of the lower-order scales “complex imagery,” “elementary imagery,” “audio-visual synesthesia,” and “changed meaning of percepts” and was used as the primary outcome to assess perceptual psychedelic effects. Two additional dimensions describe “Auditory Alterations” (15 items) and “Reduction of Vigilance” (12 items). The total 3D-ASC score is the total of the three main dimensions “Oceanic Boundlessness”, “Anxious Ego-Dissolution”, and “Visionary Restructuralization” and can be used as a measure of the overall intensity of the alteration of the mind [13]. The scale is well-validated in German [11] and many other languages and widely used to characterize the subjective effects of various psychedelic drugs. In particular, the scale has been used by most research groups to psychometrically assess LSD, psilocybin, and MDMA effects [3,4,9,14-18]. Furthermore, acute ratings on the 5D-ASC after administration of psilocybin and LSD have been used to predict long-term therapeutic effects of psychedelic treatments in patients [19-21]. Ratings on the 5D-ASC have been shown to closely correlate with ratings on the Mystical Effects Questionnaire (MEQ, see below) [13], which is primarily used by research groups in the US [20].

*Psychedelic Experience Scale (PES) and Mystical Effects Questionnaire (MEQ)*

Mystical experiences were assessed 12 h after drug administration using the Psychedelic Experience Questionnaire/Scale (PES) [22] that represents a revalidation of the original 100-item States of Consciousness Questionnaire (SOCQ) [13,23] and includes the 43-item Mystical Effects Questionnaire (MEQ43) [23], the 30-item Mystical Effects Questionnaire (MEQ30) [24], and the 40-item Mystical Effects Questionnaire (MEQ40) [22]. The MEQ30 subscales are “mystical”, “positive mood”, “transcendence of time/space”, and “ineffability” and their total provides the MEQ30 total score. Ten more items allow for derivation of the additional subscales “paradoxicality” and “connectedness” (40-item MEQ40). Eight more items allow for derivation of the additional “visual experience” and “distressing experience” subscales that together with all other subscales for the PES subscales (48 items from the 100-item SOCQ). Note that the full 100-item questionnaire was completed by the participants and only 48 items are needed to derive the validated subscales [22]. Future research could use the full 100-item scale (SOCQ) or just the 48-items needed for the PES analysis. The published German version was used [13,22]. The MEQ has been used in numerous experimental and therapeutic trials with psilocybin [20,23,25-31]. The MEQ has also been used in many experimental trials with LSD and MDMA [3,4,7,9,32,33]. We also derived the four scale scores of the validated revised 30-item MEQ: mystical, positive mood, transcendence of time and space, and ineffability [24].

*Plasma concentrations*

Blood was collected into lithium heparin tubes. The blood samples were immediately centrifuged, and the plasma was subsequently stored at -80°C until analysis. HMA and HMMA concentrations were determined after enzymatic deglucuronidation. Lys-MDMA and Lys-MDA signals were monitored and normalized to the internal standards corresponding to MDMA and MDA, respectively.

**Results**


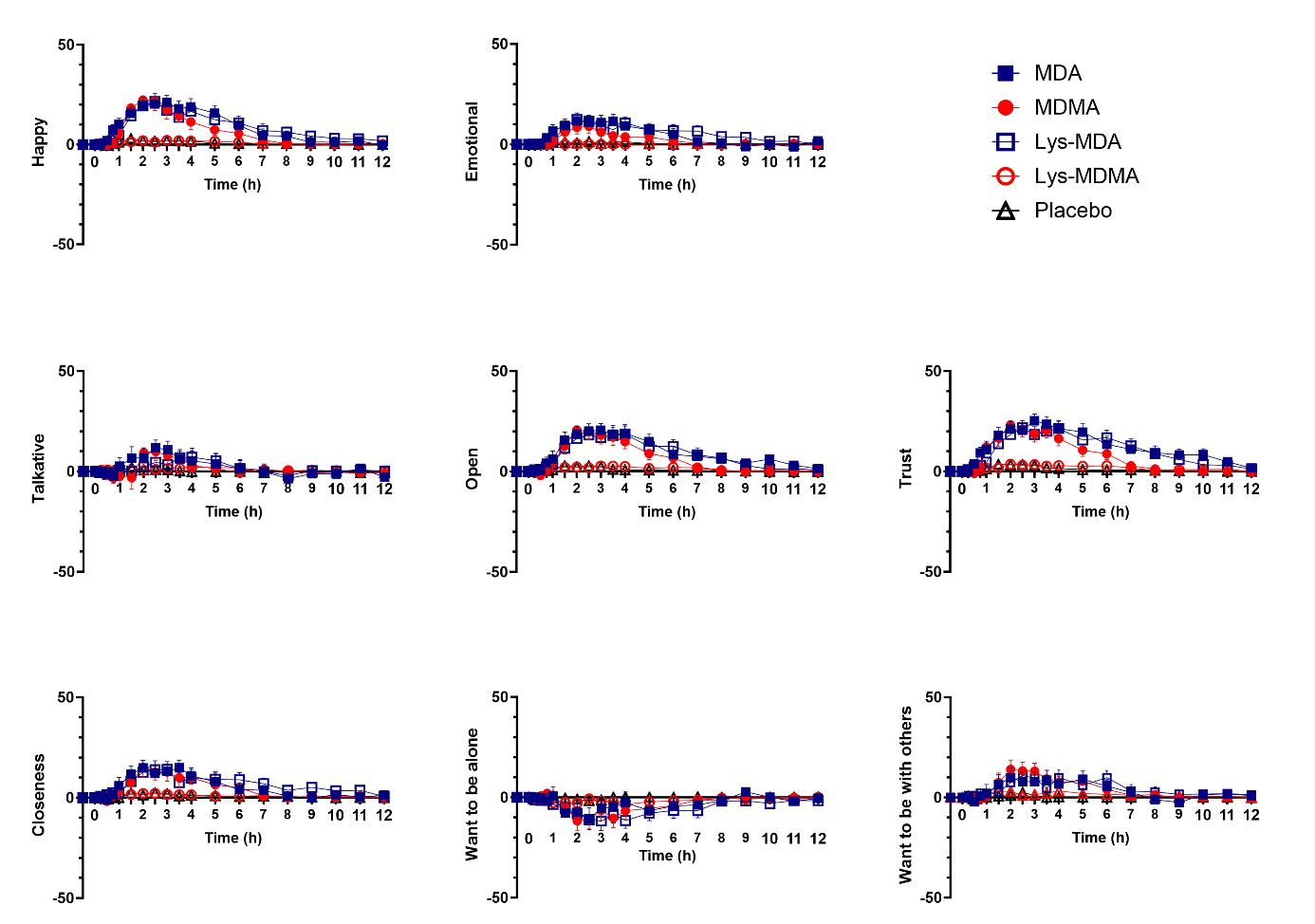


**Figure S1.** Acute subjective effects induced by 3,4-methylenedioxymethamphetamine (MDMA), 3,4-methylenedioxyamphetamine (MDA), the prodrugs Lysine-MDMA (Lys-MDMA) and Lysine-MDA (Lys-MDA), and placebo over time on the bidirectional VASs. MDMA, MDA, and Lys-MDA induced similar increases on the VASs shown in this figure. Lys-MDMA did not induce any significant subjective effects compared with placebo. MDMA, MDA, Lys-MDMA, Lys-MDA or placebo was administered at t = 0 h. The data are expressed as the mean ± SEM percentage of maximally possible scale scores in 23 subjects. The corresponding maximal responses and statistics are shown in Supplementary Table S2.


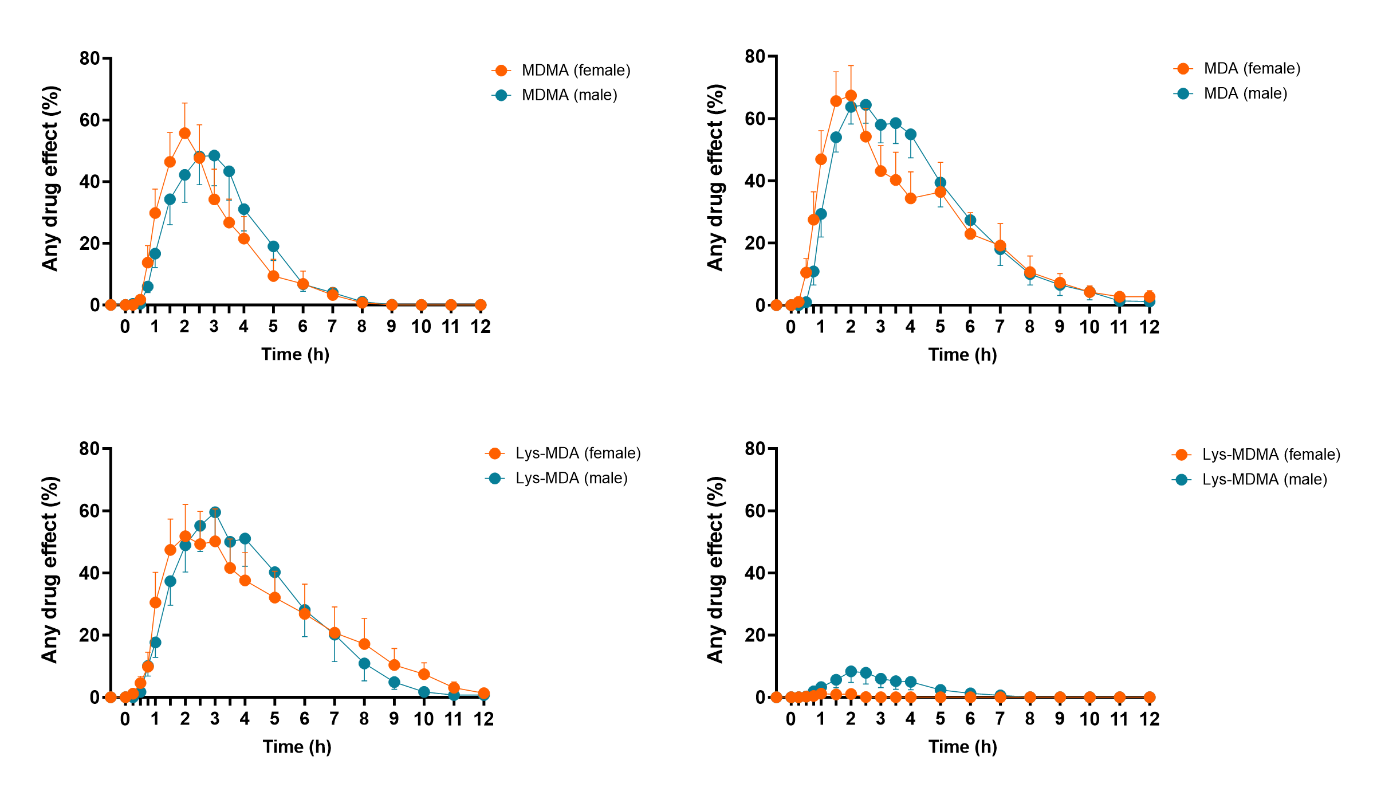


**Figure S2.** Sex differences in the VAS “any drug effects” in response to MDMA, MDA, Lys-MDA, and Lys-MDMA. Female participants reported similar “any drug effects” compared with male participants across all substance conditions. MDMA, MDA, Lys-MDA, Lys-MDMA or placebo was administered at t = 0 h. The data are expressed as the mean ± SEM percentage of maximally possible scale scores in 23 participants (12 female, 11 male). The corresponding maximal responses are shown in Supplementary Table S6.


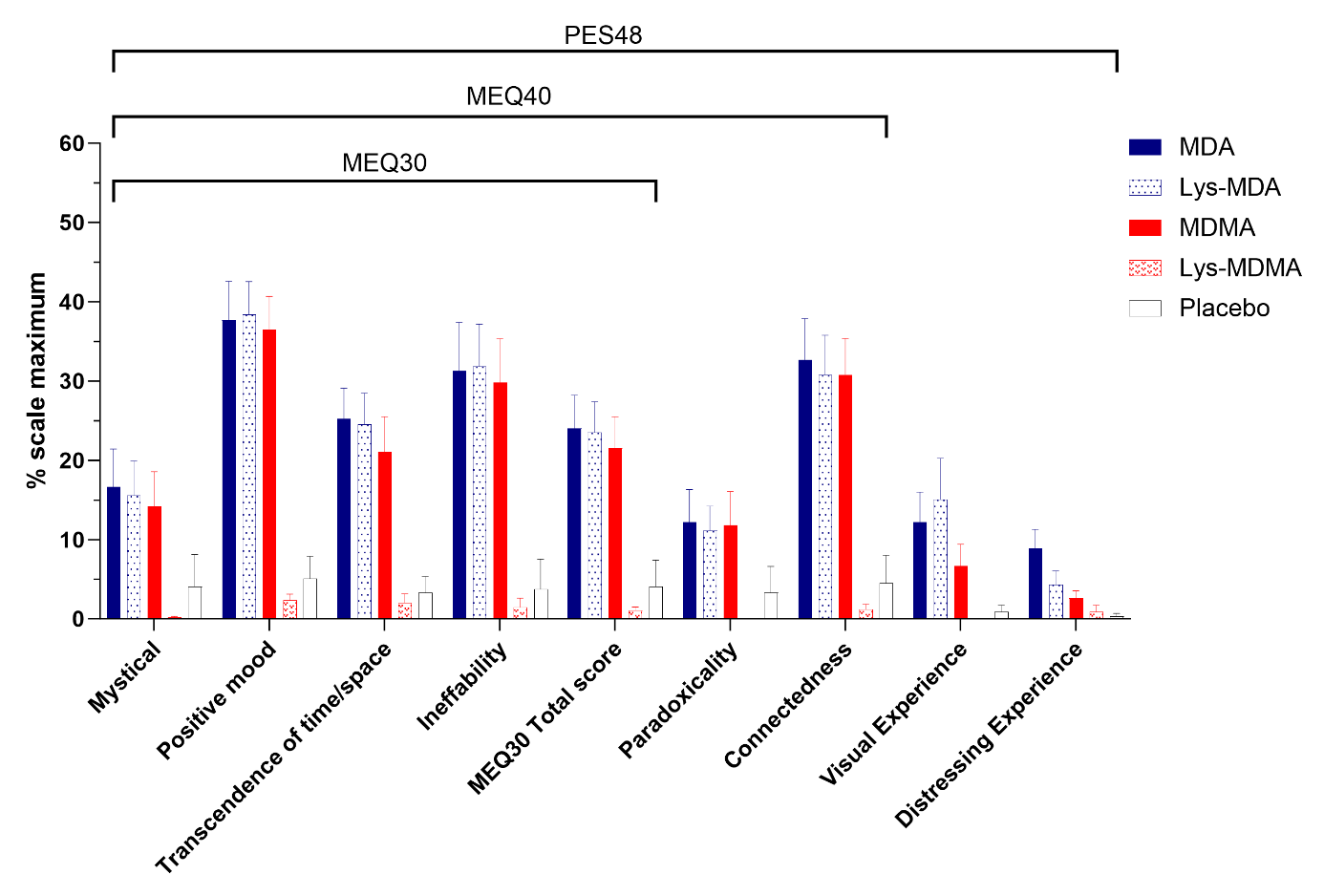


**Figure S3.** Acute mystical-type experiences on the Psychedelic Experience Scale (PES) and the 30- and 40-item Mystical Effects Questionnaire (MEQ30 and MEQ40, respectively). MDMA, MDA, and Lys-MDA induced overall comparable effects on the MEQ30, the MEQ40, and the 48-item PES48. MDA increased ratings on the subscale “distressing experience” more compared to MDMA. Lys-MDMA did not induce any effects on the PES compared to placebo.

The data are expressed as the mean ± SEM percentage of maximally possible scale scores in 23 subjects. Statistics are shown in Supplementary Table S3.


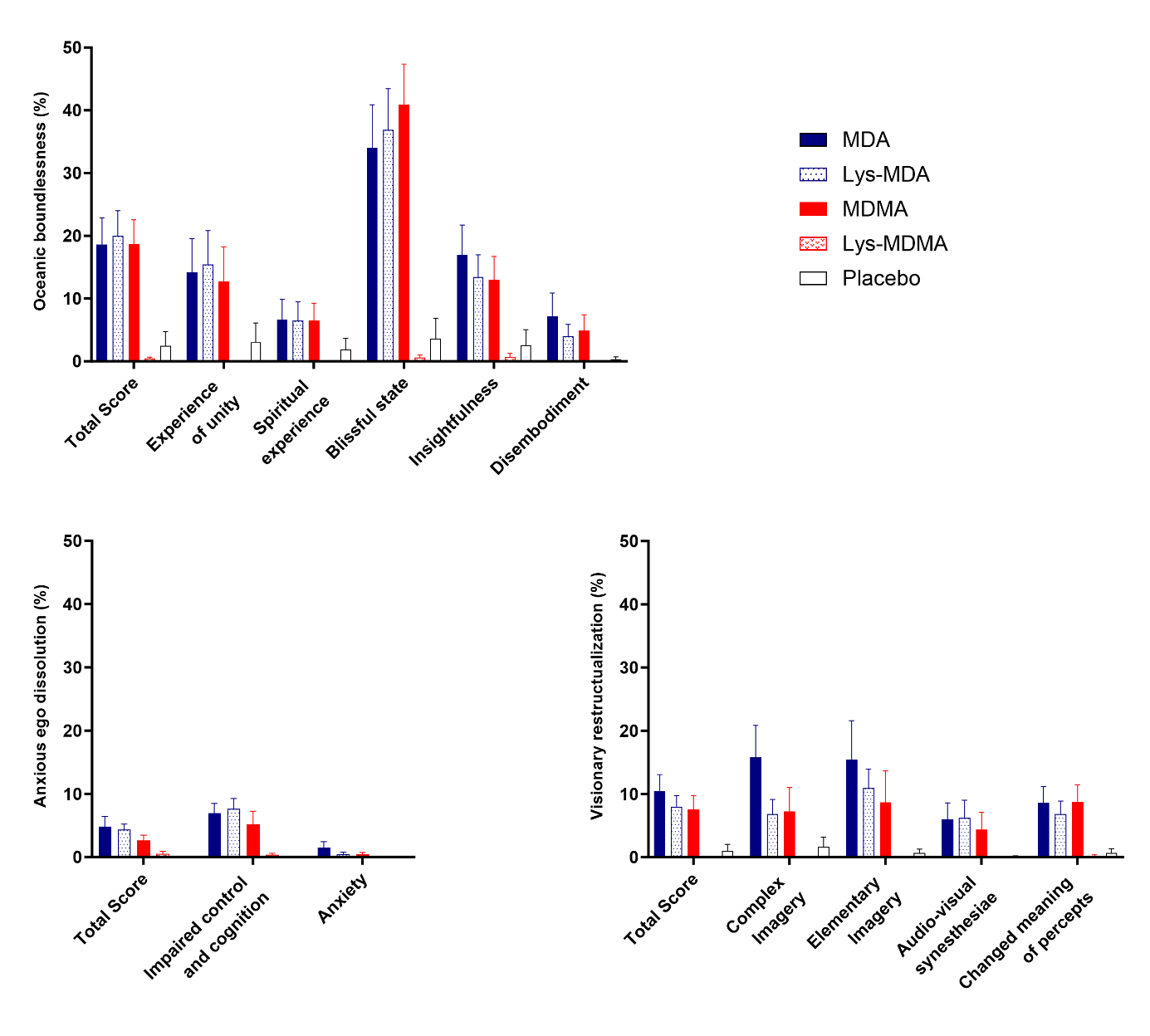


**Figure S4.** Acute mystical-type experiences on the 5 Dimensions of Altered States of Consciousness (5D-ASC) scale. MDMA, MDA, and Lys-MDA induced comparable alterations of the mind. MDMA did not induce the anxious ego dissolution total score compared to placebo and Lys-MDMA, while MDA and Lys-MDA showed a significant increase on this dimension. The administration of placebo and Lys-MDMA did not induce any significant alterations of mind. The data are expressed as the mean ± SEM percentage of maximally possible scale scores in 23 subjects. Statistics are shown in Supplementary Table S4.


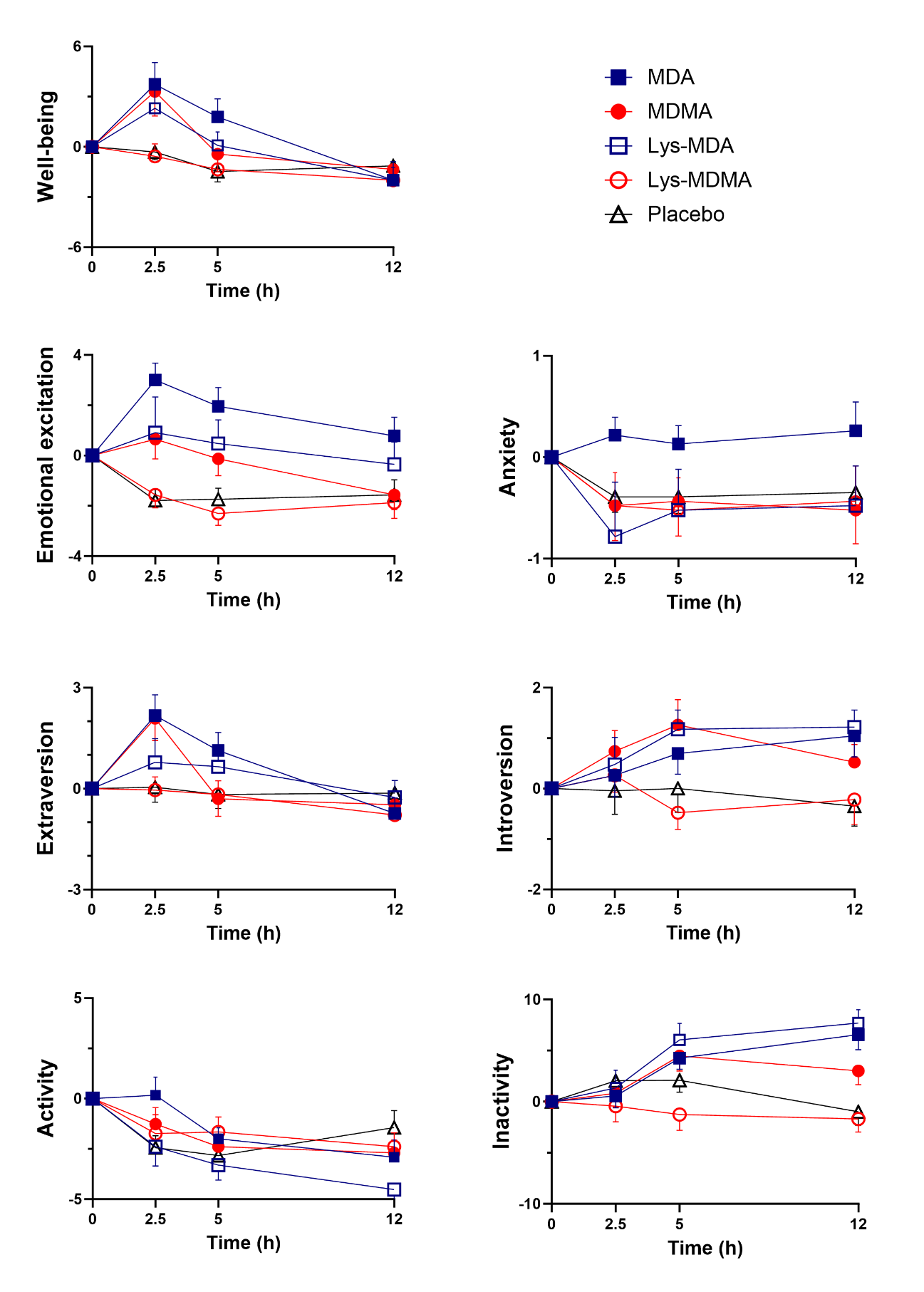


**Figure S5**. Subjective effects over time on the Adjective Mood Rating Scale (AMRS). MDMA, MDA, and Lys-MDA all similarly increased well-being, while only MDA and Lys-MDA significantly increased emotional excitation compared with Lys-MDMA and placebo. Lys-MDMA did not induce any effects on the AMRS compared to placebo. No substance induced significant changes in “introversion”, “activity”, “inactivity”, “concentration”, or “anxiety” on the AMRS. MDMA, MDA, Lys-MDMA, Lys-MDA, or placebo was administered at t = 0 h. The data are expressed as mean ± SEM changes from baseline in 23 subjects. The corresponding maximal effects and statistics are shown in Supplementary Table S5.


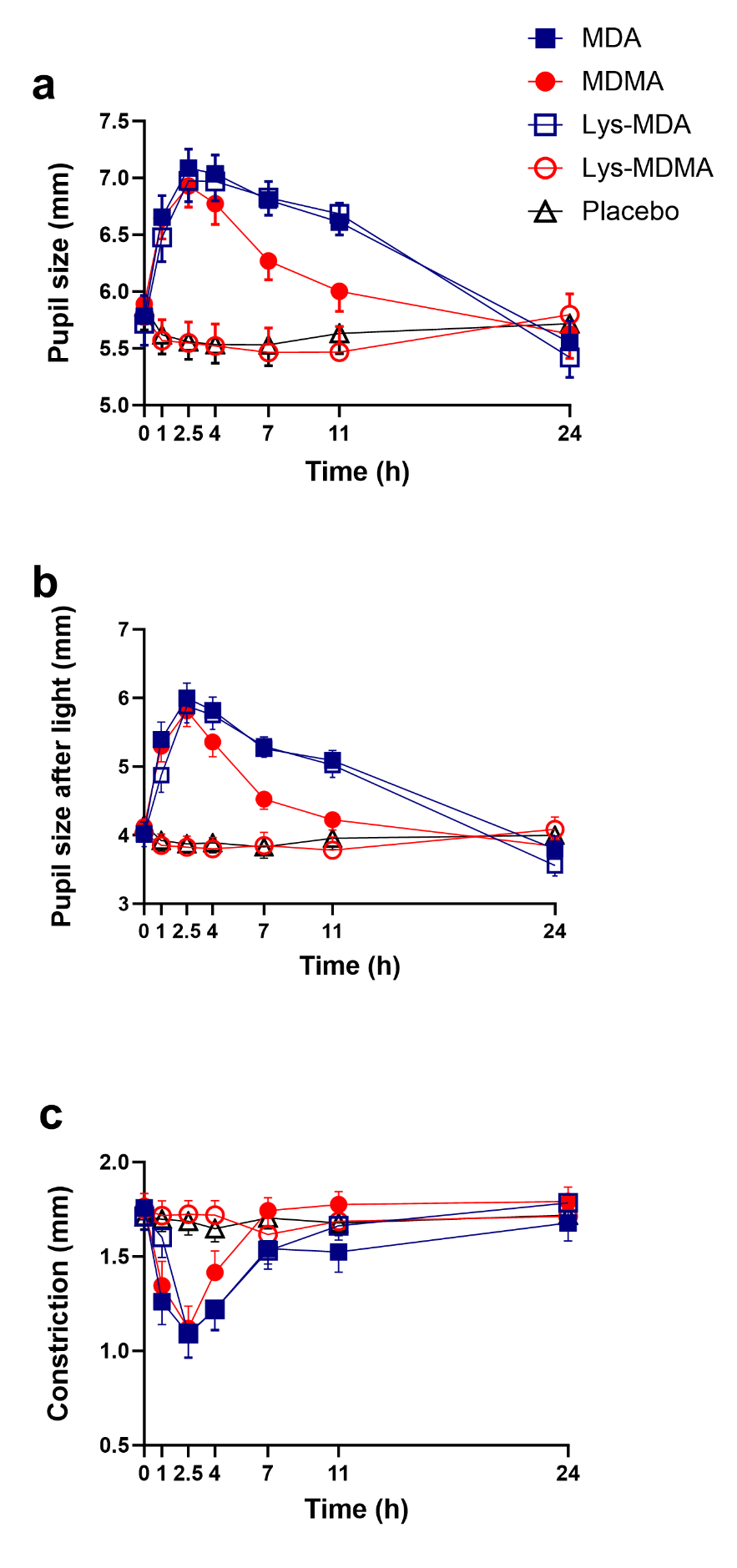


**Figure S6.** Effects of MDMA, MDA, Lys-MDMA, Lys-MDA, and placebo over time on pupillary function. MDMA, MDA, and Lys-MDA increased pupil size **(a-b)** and showed a reduced reaction to light **(b)** compared to Lys-MDMA and placebo **(c)**. MDMA, MDA, Lys-MDMA, Lys-MDA or placebo was administered at t = 0 h. The data are expressed as the mean ± SEM in 23 subjects. The corresponding maximal effects and statistics are shown in Table 1.


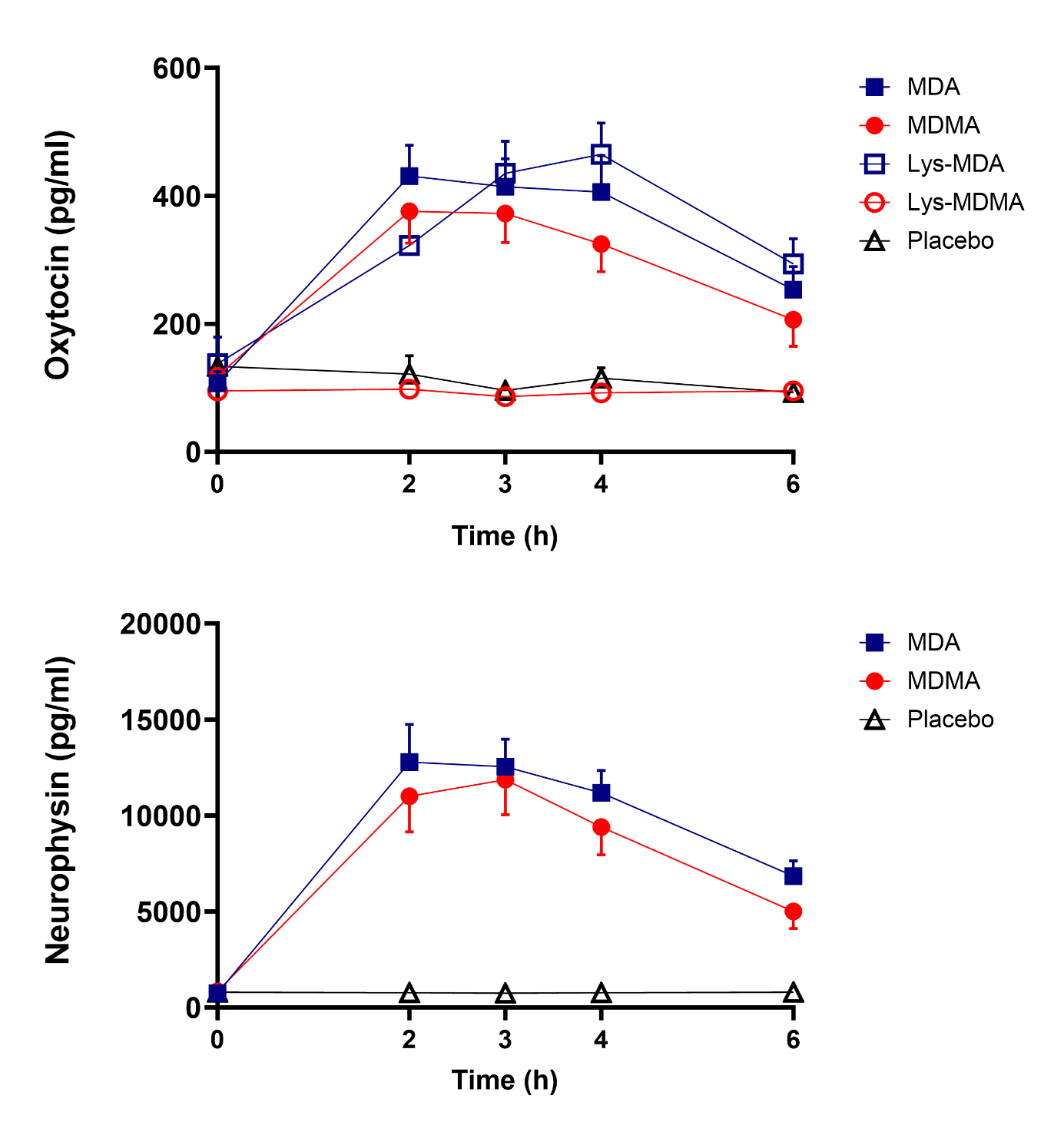


**Figure S7.** Plasma concentrations of oxytocin and its precursor neurophysin I after administration of MDMA, MDA, Lys-MDMA, Lys-MDA, and placebo. MDMA, MDA, and Lys-MDA increased oxytocin plasma concentrations compared with Lys-MDMA and placebo. MDA and MDMA increased neurophysin I plasma concentration compared with placebo. The data are expressed as mean ± SEM in 23 participants. MDMA, MDA, Lys-MDMA, Lys-MDA or placebo was administered at t = 0 h. The corresponding maximal effects and statistics are shown in Table 1.


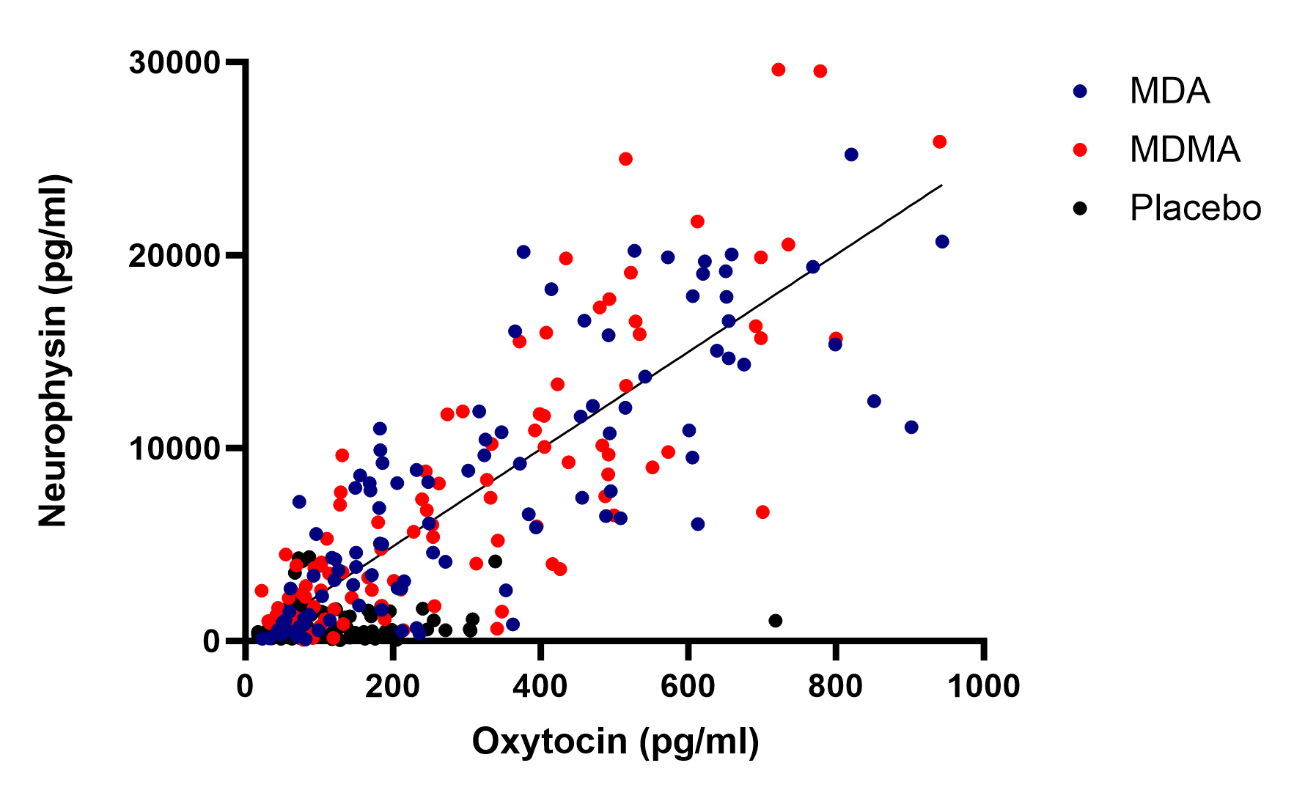


**Figure S8.** Correlation between oxytocin and neurophysin I plasma levels after administration of MDMA, MDA, and placebo. Increases in oxytocin levels significantly correlated with increases in neurophysin I. Pearson correlation coefficients and p-values are r = 0.82 and *p<0.0001*.


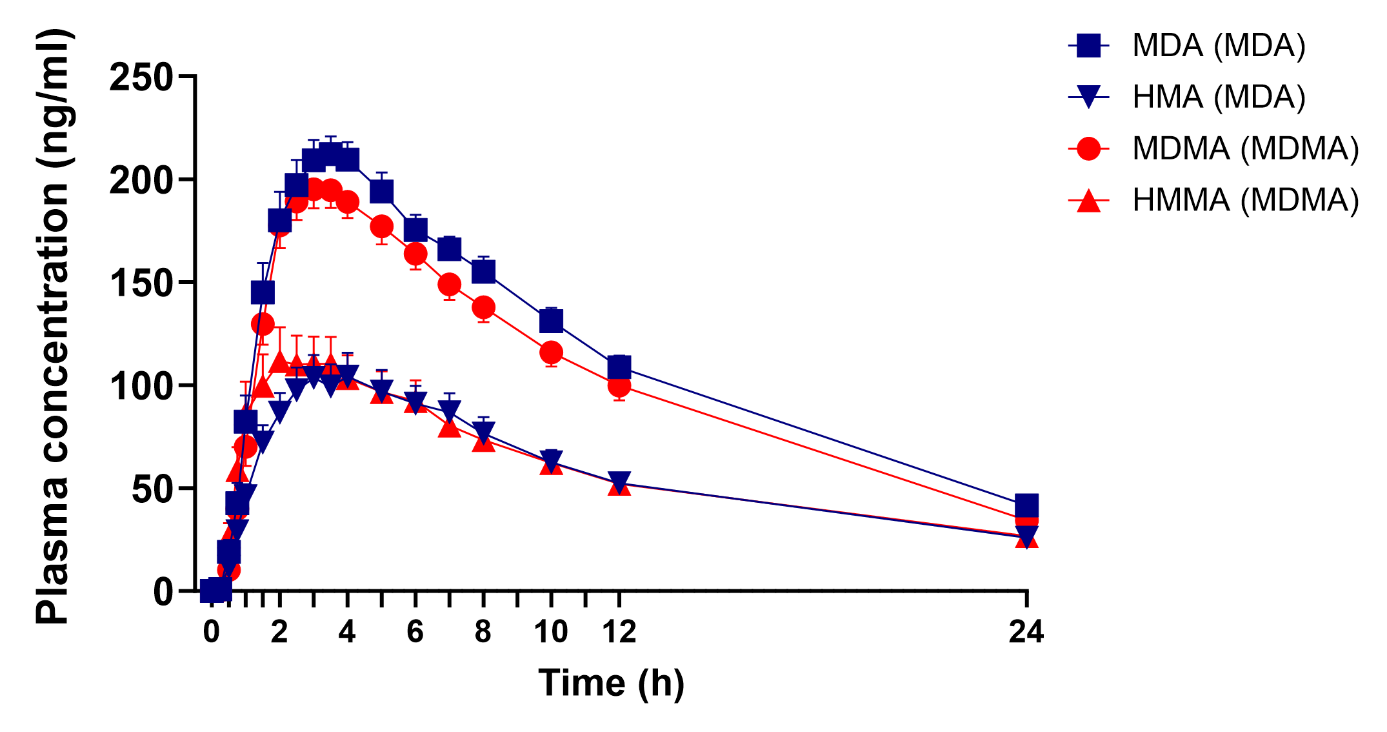


**Figure S9.** Plasma concentrations of MDA and MDMA and their respective metabolites HMA and HMMA after administration of MDA and MDMA in equimolar doses. HMMA and HMA concentrations were determined after enzymatic deglucuronidation. Plasma concentration (C_max_ and area under the curve (AUC)) was higher, and half-life (t_1/2_) was longer for MDA compared to MDMA. The data are expressed as mean ± SEM in 23 participants. MDMA or MDA was administered at t = 0 h. The corresponding pharmacokinetic parameters were determined by non-compartmental analysis and are shown in Supplementary Table S9.


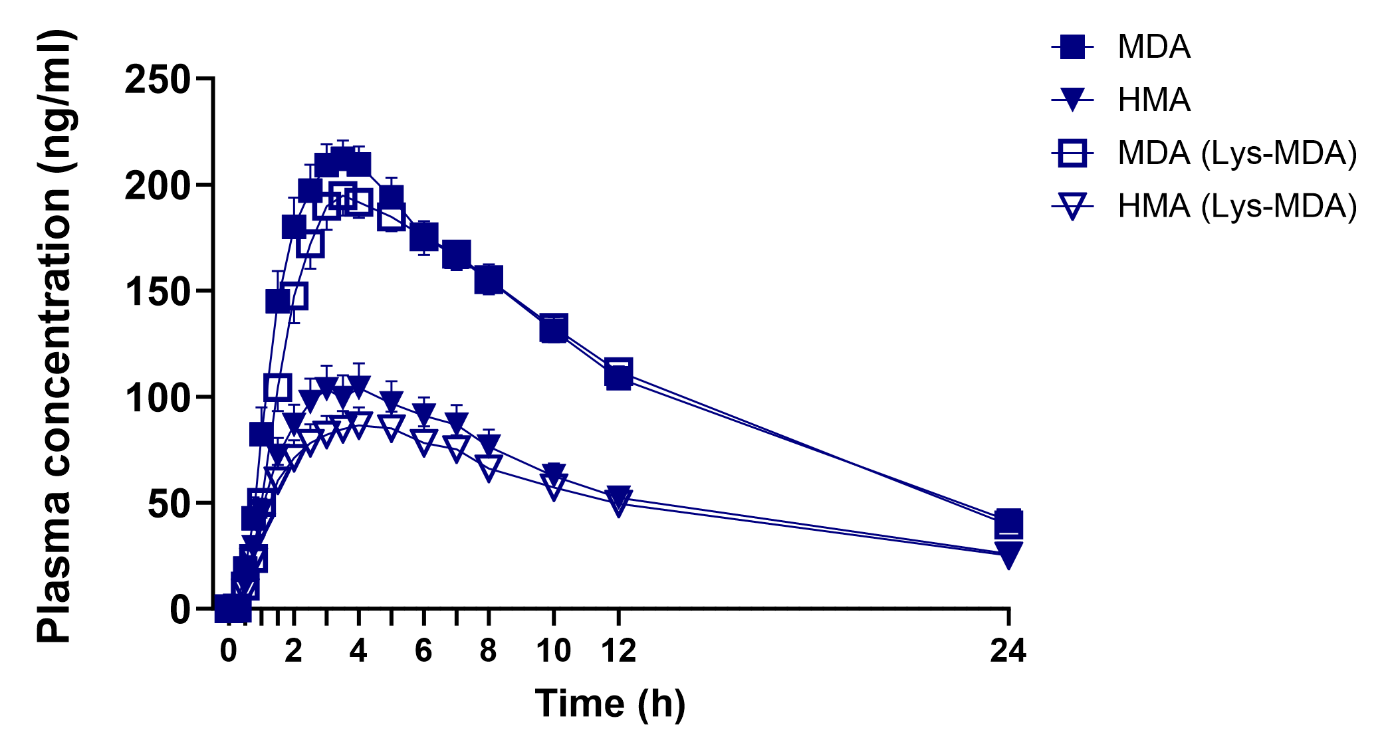


**Figure S10.** Plasma concentrations of MDA and its metabolite HMA after administration of MDA or Lys-MDA at equimolar doses. HMA concentrations were determined after enzymatic deglucuronidation. The peak plasma concentration (t_max_) occurred later and plasma concentration (C_max_ and area under the curve (AUC)) were slightly lower after administration of the prodrug Lys-MDA compared with administration of MDA. The data are expressed as mean ± SEM in 23 participants. MDA or Lys-MDA was administered at t = 0 h. The corresponding pharmacokinetic parameters were determined by non-compartmental analysis and are shown in Supplementary Table S9.


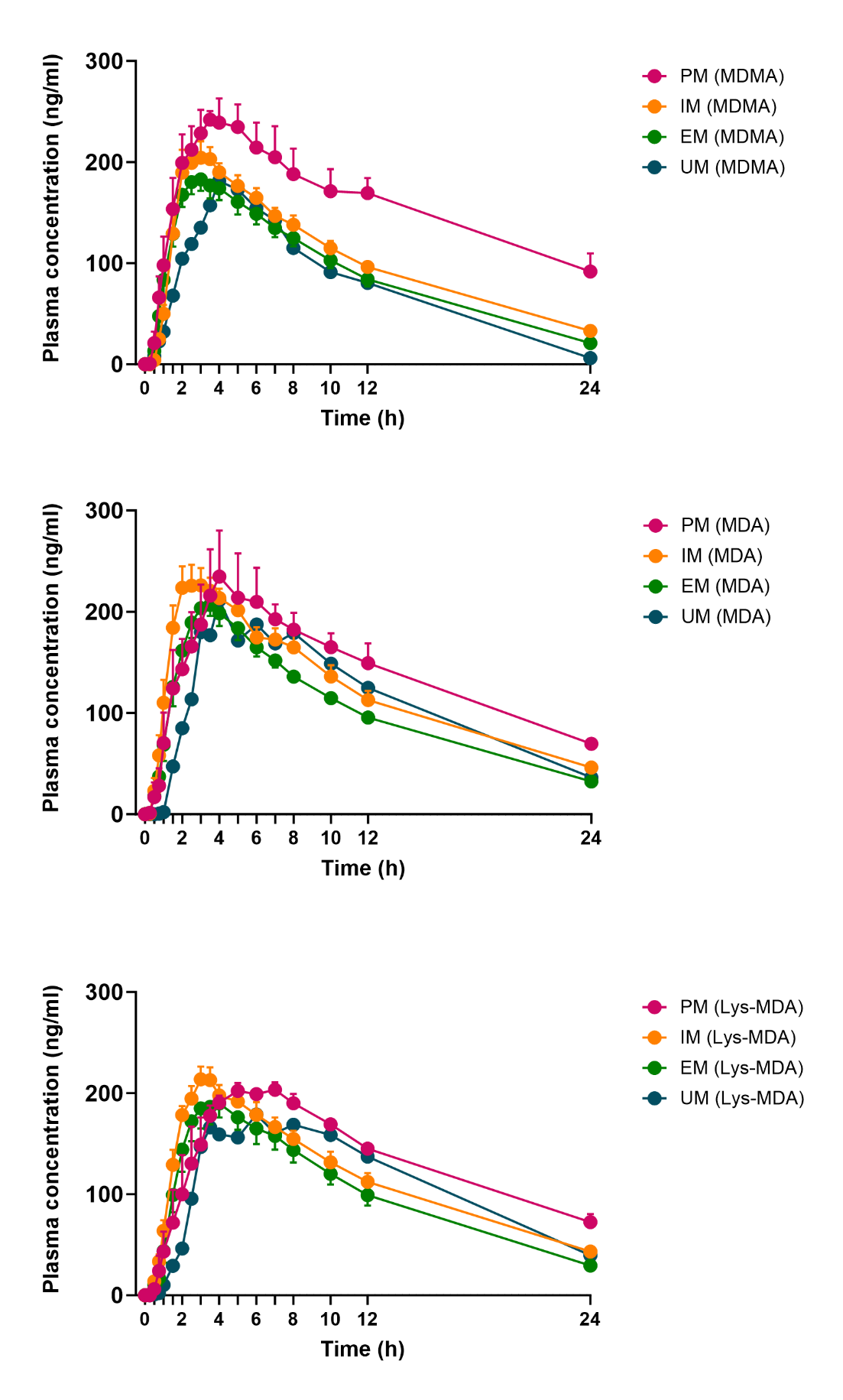


**Figure S11.** Plasma concentrations of MDMA or MDA over time in different Cytochrome P450 2D6 (CYP2D6) metabolizers when MDMA, MDA, or Lys-MDA was administered. Genotypes were classified based on genetically determined CYP2D6 enzyme activity. PM = poor metabolizer, IM = intermediate metabolizer, EM = extensive metabolizer, UM = ultrarapid metabolizer.


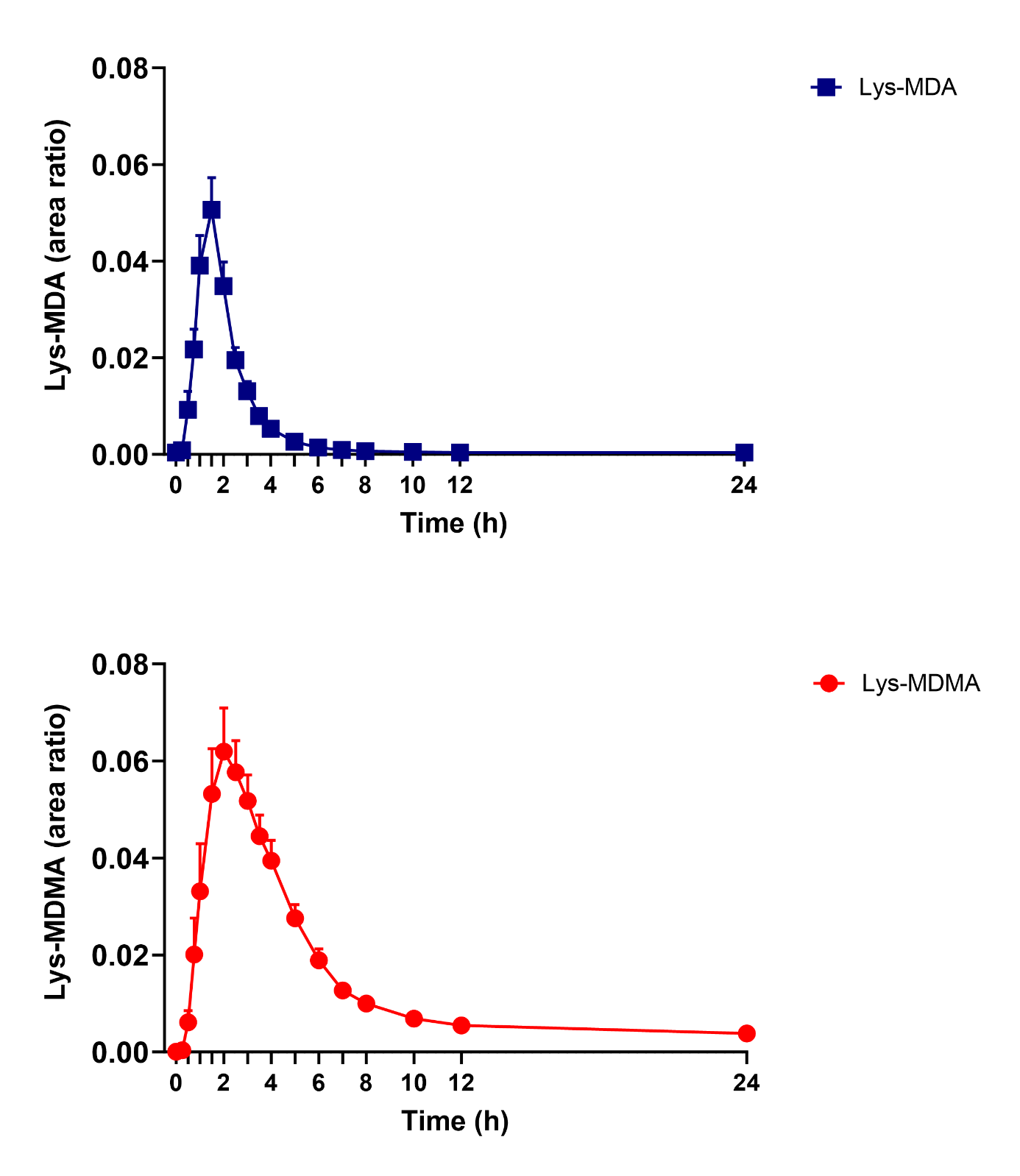


**Figure S12.** Area ratios of Lys-MDA and Lys-MDMA are presented as the signal intensities of Lys-MDA and Lys-MDMA relative to their respective internal standard, MDA-d5 and MDMA-d5. The time to reach maximal concentration (T_max_) was 1.6 ± 0.1 hours (mean ± SEM) for Lys-MDA and 2.2 ± 0.1 hours for Lys-MDMA.

*Placebo effects*

When comparing the placebo and Lys-MDMA sessions (with Lys-MDMA serving as a second unexpected placebo session), the participants reported greater subjective effects during the second later occurring session regardless of the actual drug condition. During the second placebo session, subjective ratings of “any drug effects,” “good drug effects,” “I like the effect,” and “stimulated” were significantly higher, with a trend toward higher ratings for “drug high” (Supplementary Table S13). This effect was most pronounced at the beginning of the session.

*Consort flow chart*


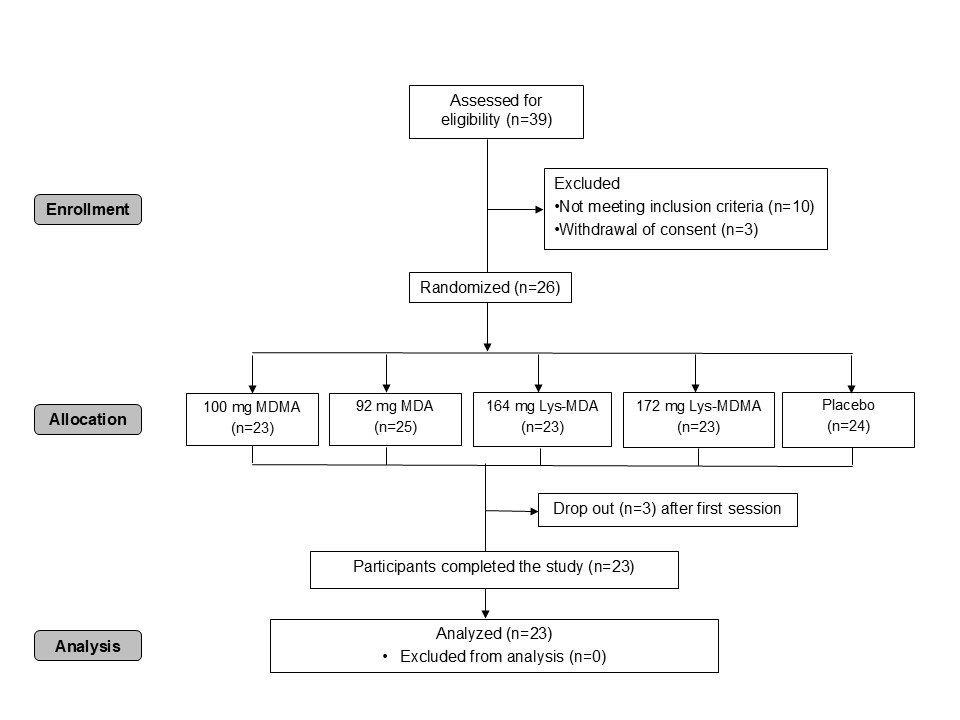


**References**

1 Wittchen H, Pfister H. (Swets Test Services, D-Frankfurt, 1997).

2 Ising HK, Veling W, Loewy RL, Rietveld MW, Rietdijk J, Dragt S, et al. The validity of the 16-item version of the Prodromal Questionnaire (PQ-16) to screen for ultra high risk of developing psychosis in the general help-seeking population. Schizophr Bull. 2012;38:1288-96.

3 Holze F, Vizeli P, Muller F, Ley L, Duerig R, Varghese N, et al. Distinct acute effects of LSD, MDMA, and D-amphetamine in healthy subjects. Neuropsychopharmacology. 2020;45:462-71.

4 Schmid Y, Enzler F, Gasser P, Grouzmann E, Preller KH, Vollenweider FX, et al. Acute effects of lysergic acid diethylamide in healthy subjects. Biol Psychiatry. 2015;78:544-53.

5 Hysek CM, Schmid Y, Simmler LD, Domes G, Heinrichs M, Eisenegger C, et al. MDMA enhances emotional empathy and prosocial behavior. Soc Cogn Affect Neurosci. 2014;9:1645-52.

6 Hysek CM, Liechti ME. Effects of MDMA alone and after pretreatement with reboxetine, duloxetine, clonidine, carvedilol, and doxazosin on pupillary light reflex. Psychopharmacology (Berl). 2012;224:363-76.

7 Straumann I, Ley L, Holze F, Becker AM, Klaiber A, Wey K, et al. Acute effects of MDMA and LSD co-administration in a double-blind placebo-controlled study in healthy participants. Neuropsychopharmacology. 2023;48:1840-48.

8 Ley L, Holze F, Arikci D, Becker AM, Straumann I, Klaiber A, et al. Comparative acute effects of mescaline, lysergic acid diethylamide, and psilocybin in a randomized, double-blind, placebo-controlled cross-over study in healthy participants. Neuropsychopharmacology. 2023;48:1659-67.

9 Holze F, Ley L, Muller F, Becker AM, Straumann I, Vizeli P, et al. Direct comparison of the acute effects of lysergic acid diethylamide and psilocybin in a double-blind placebo-controlled study in healthy subjects. Neuropsychopharmacology. 2022;47:1180-87.

10 Janke W, Debus G. Die Eigenschaftswörterliste. Hogrefe: Göttingen.; 1978.

11 Dittrich A. The standardized psychometric assessment of altered states of consciousness (ASCs) in humans. Pharmacopsychiatry. 1998;31 (Suppl 2):80-4.

12 Studerus E, Gamma A, Vollenweider FX. Psychometric evaluation of the altered states of consciousness rating scale (OAV). PLoS One. 2010;5:e12412.

13 Liechti ME, Dolder PC, Schmid Y. Alterations in conciousness and mystical-type experiences after acute LSD in humans. Psychopharmacology. 2017;234:1499-510.

14 Carhart-Harris RL, Kaelen M, Bolstridge M, Williams TM, Williams LT, Underwood R, et al. The paradoxical psychological effects of lysergic acid diethylamide (LSD). Psychol Med. 2016;46:1379-90.

15 Dolder PC, Schmid Y, Mueller F, Borgwardt S, Liechti ME. LSD acutely impairs fear recognition and enhances emotional empathy and sociality. Neuropsychopharmacology. 2016;41:2638-46.

16 Bershad AK, Schepers ST, Bremmer MP, Lee R, de Wit H. Acute subjective and behavioral effects of microdoses of lysergic acid diethylamide in healthy human volunteers. Biol Psychiatry. 2019;86:792-800.

17 Preller KH, Herdener M, Pokorny T, Planzer A, Kraehenmann R, Stämpfli P, et al. The fabric of meaning and subjective effects in LSD-induced states depend on serotonin 2A receptor activation Curr Biol. 2017;27:451-57.

18 Schmid Y, Gasser P, Oehen P, Liechti ME. Acute subjective effects in LSD- and MDMA-assisted psychotherapy. J Psychopharmacol. 2021;35:362-74.

19 Roseman L, Nutt DJ, Carhart-Harris RL. Quality of acute psychedelic experience predicts therapeutic efficacy of psilocybin for treatment-resistant depression. Front Pharmacol. 2017;8:974.

20 Griffiths RR, Johnson MW, Carducci MA, Umbricht A, Richards WA, Richards BD, et al. Psilocybin produces substantial and sustained decreases in depression and anxiety in patients with life-threatening cancer: a randomized double-blind trial. J Psychopharmacol. 2016;30:1181-97.

21 Holze F, Gasser P, Muller F, Dolder PC, Liechti ME. Lysergic acid diethylamide-assisted therapy in patients with anxiety with and without a life-threatening illness: a randomized, double-blind, placebo-controlled phase II study. Biol Psychiatry. 2023;93:215-23.

22 Stocker K, Hartmann M, Ley L, Becker AM, Holze F, Liechti ME. The revival of the psychedelic experience scale: revealing its extended-mystical, visual, and distressing experiential spectrum with LSD and psilocybin studies. J Psychopharmacol. 2024;38:80-100.

23 Griffiths RR, Richards WA, McCann U, Jesse R. Psilocybin can occasion mystical-type experiences having substantial and sustained personal meaning and spiritual significance. Psychopharmacology. 2006;187:268-83; discussion 84-92.

24 Barrett FS, Johnson MW, Griffiths RR. Validation of the revised Mystical Experience Questionnaire in experimental sessions with psilocybin. J Psychopharmacol. 2015;29:1182-90.

25 MacLean KA, Johnson MW, Griffiths RR. Mystical experiences occasioned by the hallucinogen psilocybin lead to increases in the personality domain of openness. J Psychopharmacol. 2011;25:1453-61.

26 Griffiths RR, Johnson MW, Richards WA, Richards BD, McCann U, Jesse R. Psilocybin occasioned mystical-type experiences: immediate and persisting dose-related effects. Psychopharmacology. 2011;218:649-65.

27 Griffiths R, Richards W, Johnson M, McCann U, Jesse R. Mystical-type experiences occasioned by psilocybin mediate the attribution of personal meaning and spiritual significance 14 months later. J Psychopharmacol. 2008;22:621-32.

28 Garcia-Romeu A, Griffiths RR, Johnson MW. Psilocybin-occasioned mystical experiences in the treatment of tobacco addiction. Curr Drug Abuse Rev. 2014;7:157-64.

29 Garcia-Romeu A, Davis AK, Erowid F, Erowid E, Griffiths RR, Johnson MW. Cessation and reduction in alcohol consumption and misuse after psychedelic use. J Psychopharmacol. 2019;33:1088-101.

30 Griffiths RR, Johnson MW, Richards WA, Richards BD, Jesse R, MacLean KA, et al. Psilocybin-occasioned mystical-type experience in combination with meditation and other spiritual practices produces enduring positive changes in psychological functioning and in trait measures of prosocial attitudes and behaviors. J Psychopharmacol. 2018;32:49-69.

31 Ross S, Bossis A, Guss J, Agin-Liebes G, Malone T, Cohen B, et al. Rapid and sustained symptom reduction following psilocybin treatment for anxiety and depression in patients with life-threatening cancer: a randomized controlled trial. J Psychopharmacol. 2016;30:1165-80.

32 Becker AM, Klaiber A, Holze F, Istampoulouoglou I, Duthaler U, Varghese N, et al. Ketanserin reverses the acute response to LSD in a randomized, double-blind, placebo-controlled, crossover study in healthy participants. Int J Neuropsychopharmacol. 2023;26:97-106.

33 Holze F, Vizeli P, Ley L, Muller F, Dolder P, Stocker M, et al. Acute dose-dependent effects of lysergic acid diethylamide in a double-blind placebo-controlled study in healthy subjects. Neuropsychopharmacology. 2021;46:537-44.
